# *tmap*: an integrative framework based on topological data analysis for population-scale microbiome stratification and association studies

**DOI:** 10.1186/s13059-019-1871-4

**Published:** 2019-12-23

**Authors:** Tianhua Liao, Yuchen Wei, Mingjing Luo, Guo-Ping Zhao, Haokui Zhou

**Affiliations:** 1 Institute of Synthetic Biology, Shenzhen Institutes of Advanced Technology, Chinese Academy of Sciences, Shenzhen, 518055 China; 2 Department of Microbiology, The Chinese University of Hong Kong, Hong Kong, 999077 SAR China; 3 Bio-Med Big Data Center, CAS Key Laboratory of Computational Biology, CAS-MPG Partner Institute for Computational Biology, Shanghai Institute of Nutrition and Health, Chinese Academy of Sciences, Shanghai, 200031 China; 4 CAS-Key Laboratory of Synthetic Biology, CAS Center for Excellence in Molecular Plant Sciences, Shanghai Institute of Plant Physiology and Ecology, Chinese Academy of Sciences, Shanghai, 200032 China; 5 Department of Microbiology and Microbial Engineering, School of Life Sciences, Fudan University, Shanghai, 200433 China; 6 Shanghai-MOST Key Laboratory of Health and Disease Genomics, Chinese National Human Genome Center, Shanghai, 201203 China; 7 Li Ka Shing Institute of Health Sciences, The Chinese University of Hong Kong, Prince of Wales Hospital, Hong Kong, 999077 SAR China

**Keywords:** Microbiome-wide association analysis, Topological data analysis, Population-scale microbiome, Microbiome stratification, Nonlinear association, Enterotype analysis

## Abstract

Untangling the complex variations of microbiome associated with large-scale host phenotypes or environment types challenges the currently available analytic methods. Here, we present *tmap*, an integrative framework based on topological data analysis for population-scale microbiome stratification and association studies. The performance of *tmap* in detecting nonlinear patterns is validated by different scenarios of simulation, which clearly demonstrate its superiority over the most commonly used methods. Application of *tmap* to several population-scale microbiomes extensively demonstrates its strength in revealing microbiome-associated host or environmental features and in understanding the systematic interrelations among their association patterns. *tmap* is available at https://github.com/GPZ-Bioinfo/tmap.

## Background

Microbiome-wide association studies (MWAS) capture the variation and dynamics of microbiome associated with host phenotypes or environment types [1–5]. In order to identify underappreciated but significant microbiome-associated host factors, comprehensive sample metadata of large-scale populations are usually collected [6–10]. For instance, the gut microbiomes of a healthy population were shown to be associated with hosts’ stool consistency and medication, which may confound the identification of disease-related markers [7]; and links among microbiome, metabolome, and diet of individual hosts were characterized with a crowdsourced cohort of over 10,000 citizen scientists [6]. However, these analyses failed to account for different association patterns among subpopulations [11] without methods for mapping a myriad of host phenotypes to complex microbiome profiles.

Identifying association patterns in a high-dimensional space of both population-scale host phenotypes and microbiome features poses challenges to existing analytic methods for microbiome analysis [12, 13]. Most of the currently available methods, such as *metagenomeSeq* [14], *DESeq2* [15], *Metastats* [16], *LEfSe* [17], and *MaAsLin* [18], are mainly based on either statistical test for differential microbiome abundance or linear regression to identify associated covariates. Differential abundance testing examines each microbiome feature individually without considering correlations among taxa [14–19]. Alternatively, dimension reduction methods can be used to project high-dimensional microbiome profiles to low-dimensional spaces for pattern discovery and association, such as principal coordinates analysis (PCoA) and principal component analysis (PCA) [13]. The resulted ordination axes can be utilized by linear regression to identify microbiome-associated host covariates using the *envfit* method in the *vegan* package [20]. There are also methods using distance matrix of microbiome beta-diversity for association analysis by fitting linear models to the distances or testing dissimilarities between groups, such as *adonis* and ANOSIM [21, 22]. These linear methods may not be able to capture nonlinear patterns of host-microbiome association in a high-dimensional microbiome dataset.

Stratification of population-scale human microbiome has been used to reveal subgroups of hosts with distinct microbiome configurations such as the identification of enterotypes [23]. Current methods for microbiome stratification or clustering analysis include partition-based clustering (for instance, *k*-means and *k*-medoids clustering) and Dirichlet multinomial mixture (DMM) models [11, 23–25]. However, these methods have limitations when applied to high-dimensional datasets. For example, *k*-means clustering may fail to separate nonlinear local manifold structures imbedded in a high-dimensional space [26], while the distribution assumption of DMM may not be fully met in real microbiome datasets [11]. Moreover, these two methods are unsupervised and divide microbiome samples into groups regardless of their associated metadata. Thus, to perform sophisticated stratification of population-scale microbiome samples, it is necessary to develop methods that can identify nonlinear local structures and can be supervised by host phenotypes.

Here, we present *tmap*, a method based on topological data analysis and network representation for stratification and association study of high-dimensional microbiome data. This method is motivated by using advanced large-scale data mining techniques to capture subtle and nonlinear patterns of high-dimensional datasets [27–30]. We adopted the *Mapper* algorithm for topological data analysis (TDA) [31], which has demonstrated its powerful abilities in analyzing complex biological and medical data [32–35]. This algorithm allows us to construct an informative and compact network representation of high-dimensional dataset. We developed *tmap* to extend the algorithm for microbiome study and utilize the network representation as an integrated framework for both association and stratification of population-scale microbiome data. This framework enables us to identify association of taxa or metadata within the entire network and to extract enrichment subnetworks of different association patterns. We validated the performance of *tmap* in detecting nonlinear host-microbiome association in different scenarios using synthetic microbiome data. Our method successfully identified most of the simulated nonlinear associations, which are hard to be detected with other methods (average ROC AUC of 0.95, 0.55, 0.89, and 0.63 for *tmap*, *envfit*, *adonis*, and ANOSIM respectively). Applying *tmap* to two population-scale human gut microbiome datasets of the Flemish Gut Flora Project (FGFP) [7] and American Gut Project (AGP) [6] extensively demonstrated its strengths in identifying nonlinear patterns and subpopulation enrichments of microbial taxa and in revealing microbiome stratifications associated with lifestyles. We also applied *tmap* to an even larger scale dataset of the Earth Microbiome Project (EMP) [5] to illustrate the multiscale interrelations among environment types.

## Results

**Fig. 1:**
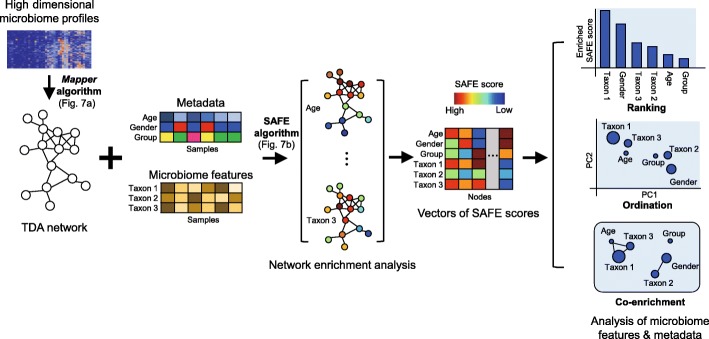
Overview of *tmap* workflow for integrative microbiome data analysis. The workflow transforms high-dimensional microbiome profiles into a compressive topological network representation for microbiome stratification and association analysis. The first step uses the *Mapper* algorithm (Fig. 2a, see the “Methods” section for details) to construct a TDA network from high-dimensional microbiome profiles. The second step uses the SAFE algorithm (Fig. 2b, see the “Methods” section for details) to map the values of metadata or microbiome features to the network to generate their vectors of SAFE scores. The last step performs ranking, ordination, and co-enrichment analysis to characterize interrelations among metadata or microbiome features based on their SAFE scores

**Fig. 2:**
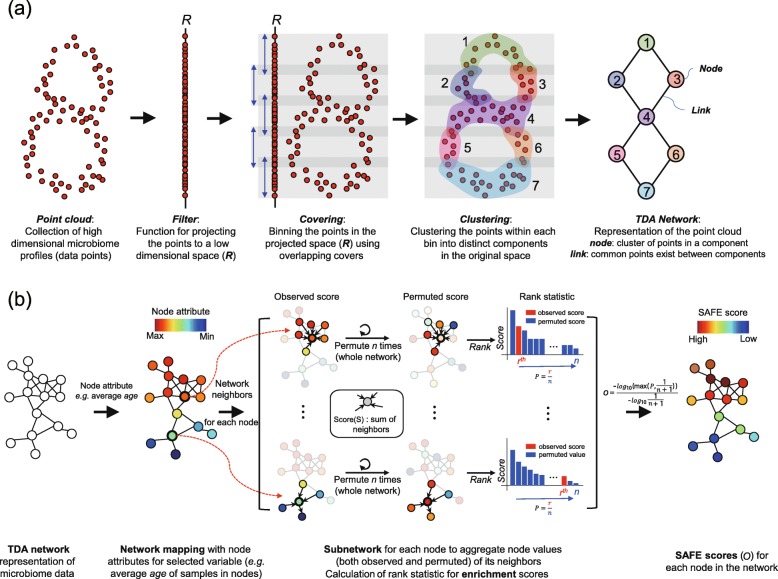
Schematic illustration of the *Mapper* and SAFE algorithms used by *tmap*. **a** The *Mapper* algorithm comprises five steps. First, data points of high-dimensional microbiome profiles (such as OTU table) are taken as input. Then, projection of the high-dimensional data points to a low-dimensional space (***R*** as shown in the figure) is performed by using a *filter* function (such as PC1 of PCoA). The *covering* step partitions the low-dimensional space into overlapping covers to bin a subset of data points within them. After that, *clustering* is conducted to cluster data points within each cover into different clusters based on their distances in the original high-dimensional space. The last step constructs a TDA network from the result of clustering analysis, in which node represents a cluster of data points and link between nodes indicates common data points between clusters. **b** The SAFE algorithm comprises three steps. Starting with a TDA network, it maps the values of metadata or microbiome features into the network as node attributes (e.g., average *age*). Second, subnetwork enrichment analysis is performed for each node to analyze its significance of the observed enrichment pattern via network permutations. This analysis is performed for each target variable (metadata or microbiome features) respectively. The last step is the calculation of SAFE score (*O*) via log transformation and normalization of the significance level of the observed enrichment. More details of these two algorithms are provided in the “Methods” section

*tmap* was developed as an integrative framework for analyzing population-scale microbiome data to identify association of host phenotypes with high-dimensional microbiome profiles and microbiome stratification enriched with host phenotypes or environment types. Underlying *tmap* is a bioinformatic pipeline of topological data analysis and network enrichment analysis for the discovery and visualization of the patterns of microbiome variation. The workflow of *tmap* consists of three major steps (Fig. 1). The first step uses the *Mapper* algorithm [31] (Fig. 2a, see the “Methods” section for details) to transform high-dimensional microbiome profiles into a TDA network to represent the microbiome variation. A node in the network represents a group of samples with highly similar microbiome profiles, and a link between two nodes indicates that common samples are shared between the two groups of samples corresponding to the nodes. Therefore, a TDA network captures both local (as local connections between nodes) and global (as global connections between nodes) patterns of microbiome variation. The second step uses a modified version of the spatial analysis of functional enrichment (SAFE) algorithm [36] (Fig. 2b, see the “Methods” section for details) to map the values of a target variable (metadata or microbiome features) into the TDA network to generate its subnetwork enrichment scores (designated *SAFE scores*, one score on each node individually). For a given target variable, such as *age*, a SAFE score on a node quantifies, statistically, the enrichment level of its values in the samples belonging to the subnetwork centered around the node (determined by a threshold of network neighborhood, see the “Methods” section for details). Together, all the SAFE scores of a target variable form a vector of values, measuring all local enrichment levels on all the nodes in a TDA network. In the last step of *tmap*, vectors of SAFE scores for each metadata or microbiome features can be compared to each other to characterize their interrelations, reflecting the similarities of their enrichment patterns in the network. This step allows us to rank driver taxa of microbiome variation, to perform ordination analysis of SAFE scores, and to calculate co-enrichment relations between metadata and microbiome features. With *tmap*, one can explore how microbiome and the host are associated at different scales, and analyze how different host factors are related to each other attributable to the corresponding microbiome variations. The utility of *tmap* framework in analyzing the datasets of synthetic microbiomes, human gut microbiomes, and the earth microbiome will be demonstrated and discussed as follows.

### Detecting nonlinear enrichment and association patterns in synthetic microbiomes

We first applied *tmap* on synthetic datasets to evaluate its ability to detect microbiome enrichment and association patterns. In order to simulate microbiomes following the distribution of species diversity and abundance in real datasets, we used *SparseDOSSA* [37] to generate synthetic microbiome data, which is a Bayesian hierarchical model and estimates species abundance parameters based on training microbiomes [37, 38]. The synthetic datasets closely resembled their training microbiome data as shown in PCoA (Bray-Curtis dissimilarity, Additional file 1: Figure S1). Based on these synthetic datasets, we simulated host factors associated with the microbiomes in different scenarios, including linear or nonlinear associations, or the mix of both of them (see the “Methods” section). After that, the performance of *tmap* in identifying the simulated associations was compared with the most commonly used methods (including *envfit*, *adonis*, and ANOSIM) to validate the advantage of *tmap* in nonlinear settings.

**Fig. 3:**
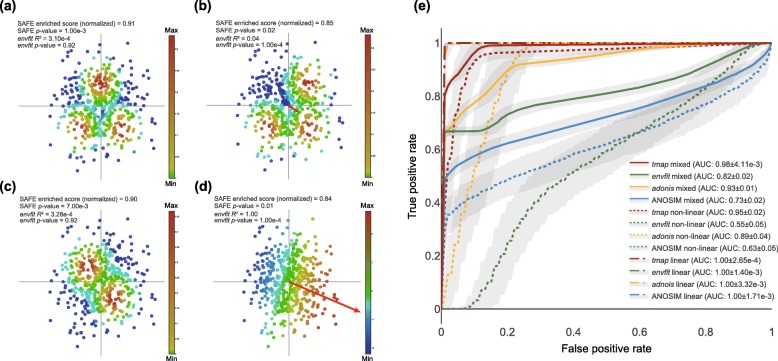
Performance of *tmap* in detecting linear and nonlinear patterns of simulated microbiome associations. Four scenarios of associations between metadata and synthetic microbiome (generated with *SparseDOSSA* [37]) are simulated. **a–d** Gaussian mixture with three symmetric centers; Gaussian mixture with three asymmetric centers; Gaussian mixture with two symmetric centers; linear association. Simulation of nonlinear associations is based on mapping the Gaussian mixtures to the first two PCs of the PCoA (principal coordinates analysis) of synthetic microbiome. Linear associations between metadata and synthetic microbiome are simulated based on linear function of the first two PCs. Arrow indicates a linear projection of the values of simulated metadata (scaled by *R*-squared using *envfit*). Significance levels and effect sizes of *envfit* (*p* value and *R*^2^) and *tmap* (*p* value and SAFE enriched score) are depicted. SAFE enriched scores are normalized (divided by the sum of SAFE scores). Color legend (from blue to red) indicates values of metadata (from small to large). **e** Receiver operating characteristic (ROC) curves of the performance of *tmap* (red) and *envfit* (green), *adonis* (yellow), and ANOSIM (blue) in detecting microbiome-associated metadata. Three scenarios of association are examined, including linear-only (dash-dot line), nonlinear-only (dotted line), and a mix (solid line) of both. The shaded areas indicate 95% confidence intervals (100 repeats). Performance is measured by ROC AUC (mean ± sd) for each method and simulation

In the case of detecting linear associations, *tmap* and the other methods in comparison exhibited similar performance (average ROC AUC of 1.00 for all of them, Fig. 3d, e). We further evaluate the performance of *tmap* in detecting nonlinear associations via simulating different nonlinear enrichment patterns in a microbiome landscape. Both symmetric and asymmetric multiple (two or three) local enrichments were included in our simulation for the detection of nonlinear associations (Fig. 3a–c). The organization of the centers of the local enrichments in each simulated association determines the strength of either linear or nonlinear effect of the association. For instance, an association pattern of symmetric multiple local enrichments can be detected by nonlinear methods, while the projection of this pattern to any linear direction will result in an averaged linear effect of almost zero (Fig. 3a, c). In contrast, asymmetric multiple local enrichments can be detected by nonlinear methods and also by linear methods because of the averaged linear effect greater than zero (Fig. 3b). Different performances in detecting the simulated nonlinear associations were observed for the four methods (average ROC AUC of 0.95, 0.55, 0.89, and 0.63 for *tmap*, *envfit*, *adonis*, and ANOSIM respectively, Fig. 3e, Additional file 20: Table S1, Additional file 2: Figure S2). Although *adonis* achieved an average ROC AUC of 0.89, which is the best among the three methods in comparison, *tmap* still significantly improved upon *adonis* (average AUC of 0.95, *p* value = 8.11e^−29^, Additional file 2: Figure S2). The weaker performance of *adonis* and the other two methods is most likely due to the linear regression technique used by these methods [20–22], which can only identify linear association between host factors and microbiome variation. In contrast, *tmap* is based on network enrichment analysis, which can analyze both linear and nonlinear associations. At last, we evaluated all the methods in a more realistic scenario that consists of both linear and nonlinear associations (see the “Methods” section). Like the above nonlinear-only scenario, *tmap* still had a significantly better outcome in this mixed scenario (average ROC AUC of 0.98, 0.82, 0.93, and 0.73 for *tmap*, *envfit*, *adonis*, and ANOSIM respectively, Fig. 3e, Additional file 20: Table S1, Additional file 2: Figure S2). Similar improvement by *tmap* over the other three methods was observed when we varied the number of simulated metadata associated with the microbiome (Additional file 2: Figure S2). Overall, *tmap* can detect both linear and nonlinear microbiome associations with comparable performance, based on network enrichment analysis rather than linear regression. As validated in the synthetic microbiomes, our method is capable of detecting various kinds of association microbiome patterns. Moreover, in addition to the patterns of multiple local enrichment, *tmap* is also capable of detecting other types of nonlinear patterns, such as circular or spiral enrichments (Additional file 19: Text S1, Additional file 3: Figure S3).

### Improving identification of human gut microbiome stratifications associated with host covariates

Host factors may be associated with gut microbiome in different ways, local or global, linear or nonlinear. In the study of the Flemish Gut Flora Project (FGFP), it was shown that only about 7.63% of the microbiome variation can be explained by the identified host covariates using linear association models [7]. *tmap* was applied to analyze the FGFP cohort data aiming at improving the identification and interpretation of such population-scale microbiome-host associations, especially in discerning nonlinear and local patterns.

**Fig. 4:**
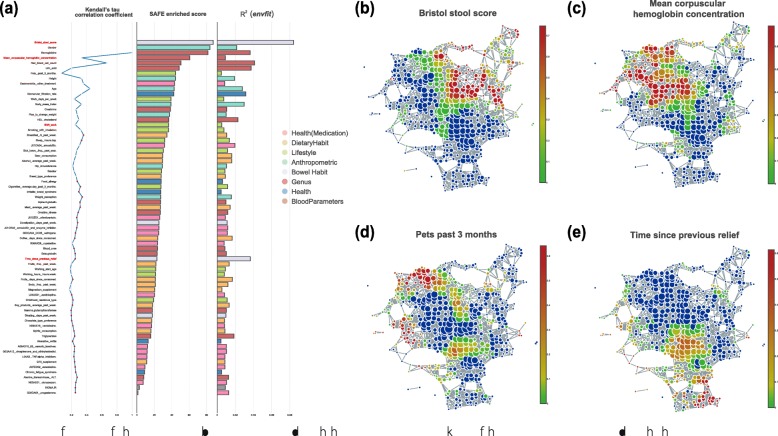
Stratification of the FGFP microbiomes associated with host covariates. **a** Ranking of host covariates associated with the FGFP microbiomes. The ranking is compared between *tmap* (middle panel, according to SAFE enriched score) and *envfit* (right panel, according to squared correlation coefficient). In the left panel, covariates that are statistically consistent between the two rankings are colored blue (Kendall’s tau, cutoff *p* value = 0.05). In the middle panel, covariates are colored based on metadata category. **b**–**e** TDA network enrichment patterns (SAFE scores) of the covariates of Bristol stool score, mean corpuscular hemoglobin concentration, pets past 3 months, and time since previous relief, respectively. Node color is based on SAFE scores of corresponding covariates, from red (large values) to blue (small values). The scale of enrichment of mean corpuscular hemoglobin concentration appears to be comparable to that of Bristol stool score, and both are ranked among the top five covariates. Nonlinear patterns of multiple local enrichments are observed for pets past 3 months and time since previous relief, which are ranked differently between *tmap* and *envfit*

Overall, host covariates identified by *tmap* showed a consistent overlap with that identified by *envfit*, particularly for the covariates ranked in the top 19 (Kendall’s tau, cutoff *p* value = 0.05, *R*^*2*^ = 0.50, Fig. 4a). Like that in the original study, Bristol stool scale (BSS) score is the top associated covariate identified by *tmap*. However, there were differences in the ranking of several host covariates, including time since previous relief, mean corpuscular hemoglobin (MCH), and shift work (Fig. 4a). We speculate that the difference might be due to nonlinear association patterns that are captured by *tmap* but not by *envfit*. The observed differences were also supported by *adonis* and ANOSIM, except for time since previous relief, which showed a weak and non-significant association with the microbiomes in ANOSIM (*R* value = 0, *p* value = 0.10, Additional file 4: Figure S4, Additional file 21: Table S2). We also noticed that ANOSIM was unable to detect most of the host covariates (only six among the total 69 covariates, cutoff *p* value = 0.05, FDR corrected [39]) that are found to be significantly associated with the microbiomes by all the other methods (67 of total 69 covariates, cutoff *p* value = 0.05, FDR corrected, Additional file 21: Table S2). As shown in the TDA network, we found that the enrichment scores of time since previous relief are lower compared to other highly enriched covariates (Fig. 4e). These low scores can be explained by the large variance of time since previous relief observed among samples in the local subnetworks (Additional file 5: Figure S5). On the other hand, enrichment scores of MCH are comparable to that of BSS, consistent with their ranking by *tmap* although the ranking of MCH by *envfit* is much lower (Fig. 4b, c).

Associations identified by *tmap* can be further stratified into subgroups in the microbiome landscape to characterize subpopulation-specific microbiome features. For instance, pet past 3 months appeared to be enriched within two subgroups (Fig. 4d), characterized by different enriched genera. *Salmonalla* and *Yersinia* were found to be enriched in the first group, whereas *Anaerofustis* and *Acetanaerobacterium* were enriched in the second group (Additional file 22: Table S3). These observations are supported by previous studies, in which *Salmonalla* and *Yersinia* were often isolated from healthy cats and dogs [40]. *Anaerofustis* and *Acetanaerobacterium* were also found in healthy pets and human gut, but their influence is yet to be understood [41, 42]. Taken together, *tmap* allows the identification of host covariates with multiple enrichment subgroups and their related microbiome features, which may help to explain microbiome variability among subpopulations and identify specific biomarkers for disease diagnosis and treatment.

### Systematic characterization of interrelations between host covariates and microbiome taxa based on more efficient stratification and association analyses

**Fig. 5:**
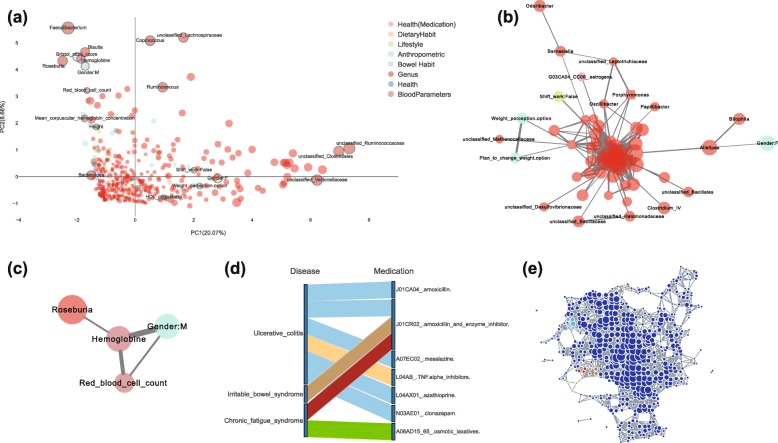
Systematic analysis of interrelations between taxa and host covariates of the FGFP microbiomes. **a** PCA (principal component analysis) of the SAFE scores of taxa and host covariates shows the overall pattern of their associations with microbiome. The top 10 covariates and taxa identified by SAFE enriched scores are highlighted (markers with edge color of gray) and annotated with their names. Host covariates are colored based on metadata category, and taxa are in red. Marker size is scaled according to the SAFE enriched score of metadata or taxa. **b**, **c** Co-enrichment networks of gender and other co-enriched host covariates and taxa, for female and male respectively. The networks reveal the interrelations between gender and other covariates or taxa when considering their associations with the FGFP microbiomes. Edge width of the network is scaled according to the negative log-transformed *p* value of Fisher’s exact test of co-enrichment. Color and size of the nodes are the same as that of PCA plot. **d** Co-enrichments between disease and medication. For instance, ulcerative colitis is co-enriched with six different drugs. On the other hand, amoxicillin and enzyme inhibitor (J01CR02) is co-enriched with three different diseases. Colors are based on their co-enrichment subnetworks. **e** Subnetworks of disease-medication co-enrichments. The identified co-enrichments are highlighted in the TDA network of the FGFP microbiomes with different colors. Co-enrichment relations of a same color indicates that they are co-enriched in a same subnetwork

Systematic characterization of interrelations among host factors correlated to the gut microbiota is valuable in understanding host-microbiome interaction. By transforming the values of host factors into SAFE scores, we were able to quantify the relations between host factors and taxa. Principal component analysis showed that the overall enrichment patterns (represented by SAFE scores) are explained mainly by the top genera as identified by SAFE enriched scores, including *Faecalibacterium*, unclassified *Ruminococcus*, and *Bacteroides* (Fig. 5a, Additional file 23: Table S4). Therefore, mapping the taxa abundance to the TDA network (by SAFE scores of taxa) allows us to identify driver species that contribute to microbiome variation and to understand how they are related to each other by PCA. This analysis also confirmed the important host covariates identified in the original study, such as gender, hemoglobin, time since previous relief, and HDL cholesterol (Fig. 5a, Additional file 6: Figure S6). We further analyzed their interrelations via co-enrichment network analysis of their SAFE scores (see the “Methods” section). The result showed that male was co-enriched with *Roseburia*, *Blautia*, *Faecalibacterium*, and hemoglobin, whereas female was co-enriched with unclassified *Bifidobacteriaceae*, unclassified *Bacillales*, *Alistipes*, and HDL cholesterol (Fig. 5b, c). These results are consistent with the well-documented facts that serum hemoglobin concentration in healthy male is often higher than female, whereas healthy female tends to have higher HDL cholesterol [43, 44]. By including both taxa and the related host factors in co-enrichment network analysis, a systematic view can be obtained to illustrate the association of blood parameters and gender with the gut microbiome, which would be valuable for understanding their possible interaction or confounding effect.

Disease and medication have been found to contribute significantly to gut microbiota variation [45, 46]. With *tmap*, we also explored how disease, medication, and microbiome would relate to each other in the FGFP cohort. The original study only found that β-lactam antibiotic caused significant increase in the abundance of specific genera. Our analysis identified seven out of the 13 drugs significantly co-enriched with three diseases, along with different associated microbiomes (Fig. 5d, e). For instance, both osmotic laxatives and β-lactam antibiotic were co-enriched with chronic fatigue syndrome, but with distinct subnetworks of the microbiome (Fig. 5d, e), highlighting the possibility of drug-specific microbiome response. We also found that commonly used drugs for ulcerative colitis (UC), such as the anti-inflammatory drug, immune system suppressors, and β-lactam antibiotics were co-enriched with UC, and are associated with different microbiomes. These results demonstrated that *tmap* may improve systematic and integrative analysis of microbiome and host phenotypes based on more efficient stratification and association methods.

### In-depth stratification of human gut microbiome associated with country and lifestyle

**Fig. 6:**
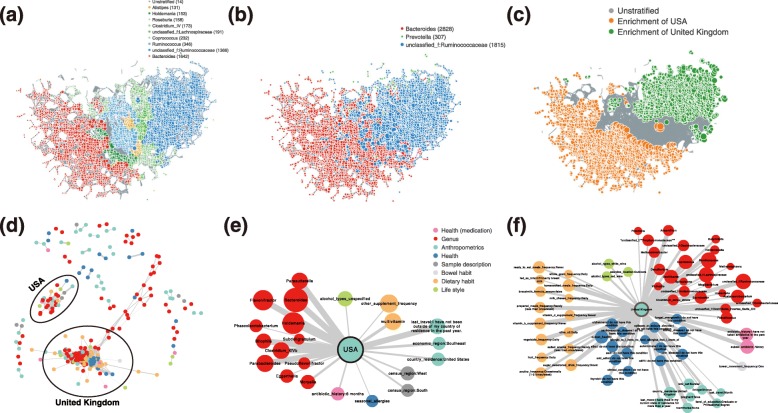
In-depth analysis of enterotype-like stratification of the AGP microbiomes and association with lifestyles. **a** Stratification of the AGP microbiomes based on enriched taxa. For each node in the TDA network, the most enriched taxon among all taxa is identified according to SAFE enriched score. Each node is colored according to its most enriched taxon. Only taxa enriched in more than 100 nodes are highlighted. Remaining unstratified nodes (with no enriched taxa) are colored in gray. **b** Stratification based on traditional enterotype analysis. Nodes are colored according to enterotype driver taxa. **c** Stratification based on countries (USA or UK). Not enriched (or unstratified) nodes are colored in gray. The number in the color legend indicates the number of nodes in the corresponding stratification. **d–f** Co-enrichment networks of lifestyle factors and taxa. Co-enrichments with countries (USA or UK) are highlighted and extracted. The extracted co-enrichment subnetworks reveal that different lifestyle factors are interrelated to the two countries when accounting for the AGP microbiomes. Node colors are based on metadata category. Node size and edge width are the same as that of Fig. 5

Human gut microbiome from different countries was shown to form clusters of distinct community compositions, which were proposed as enterotypes [23]. As a means of stratification of human gut microbiome, clustering approach has been used for enterotype analysis to identify microbiome configurations with distinct responses to drugs or diets [11, 23, 47]. Alternatively, *tmap* provides another stratification approach based on enrichment patterns of taxa abundance. We applied both approaches to the microbiome data from the American Gut Project (AGP), which comprises microbiome samples from over 10,000 citizen scientists [6]. The results showed that both approaches were able to reveal a global pattern of stratifications in the microbiome landscape, driven by different taxa (Fig. 6a, b). In addition, *tmap* also detected local in-depth stratifications of samples and their driver taxa (Fig. 6a). For instance, the Firmicutes enterotype (ET F) was subdivided into several local stratifications enriched with different taxa of Firmicutes, including *Roseburia*, *Clostridium* cluster *IV*, unclassified *Lachnospiraceae*, *Coprococcus*, *Ruminococcus*, and unclassified *Ruminococcaceae* (Additional file 24: Table S5). In contrast to enterotype stratification requiring the number of clusters to be set before analysis [23], *tmap* automatically identifies stratifications of samples based on taxa SAFE scores and the TDA network of microbiome. Our approach to stratifying microbiome variations according to the enrichment patterns of taxa can help to alleviate the problem of inferring discretized enterotypes from the continuous changes of microbiome taxa [48]. Further examination of these ET F local stratifications revealed their associations with host covariates. Existence of ET F local stratifications was also observed in the FGFP microbiomes (Additional file 7: Figure S7). This implies that augmenting known enterotypes with these local stratification patterns will further dissect population-scale microbiome variations for the identification of stratification-specific microbiome markers and their links with host phenotypes.

Stratification of a population-scale microbiome could be attributed to interactions between host phenotypes and the gut microbiota [11]. We performed ordination analysis of the SAFE scores to reveal the interrelations between the host covariates and taxa accounting for the variation of the AGP microbiomes (Additional file 8: Figure S8, see the “Methods” section). Two of the most prominent host covariates are countries (USA or UK), which were co-enriched with different microbial taxa (Fig. 6c). For instance, USA samples were co-enriched with *Bacteroides*, whereas UK samples were co-enriched with unclassified *Ruminococcaceae*. These co-enriched taxa have also been identified in the above enterotype and stratification analysis, indicating that the stratification is most likely associated with countries. As reported in previous studies, *Bacteroides* is an enterotype-driven genus and has been associated with a carnivorous dietary habit [23, 47]. With the available metadata on host lifestyles and dietary habits, we also performed co-enrichment network analysis based on their SAFE scores (see the “Methods” section). The resulted networks showed that most of the host factors and taxa were co-enriched with two hubs, corresponding to the two countries (Fig. 6d–f). The co-enrichments reflect different lifestyles associated with the two countries. For instance, UK samples were co-enriched with homecooked meals frequency (daily), milk cheese frequency (daily), whole grain frequency (daily), and vegetable frequency (daily). On the other hand, USA samples were co-enriched with antibiotic history (6 months), multivitamin, and unspecified alcohol types. Together, the co-enrichment networks indicate that the stratification of the AGP microbiomes can be further linked to lifestyles associated with different countries. This analysis demonstrated the strength of *tmap* in providing an integrative framework both for stratifying microbiomes and for illustrating the interrelations among host factors contributing to the stratification.

### Illustrating the multiscale pattern of the earth microbiome and environment types

**Fig. 7:**
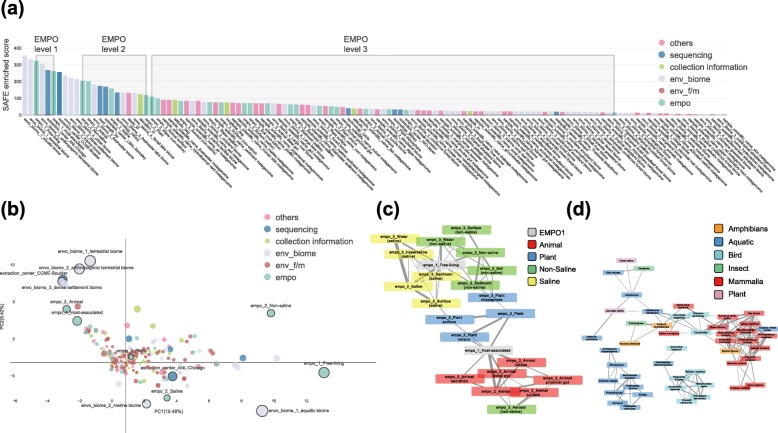
Systematic characterization of the multiscale pattern of environment types associated with the Earth’s microbiomes. **a** Ranking of EMPO, ENVO, and other metadata based on SAFE enriched score. Metadata is colored based on their categories. The relative order of EMPO classes among the ranking is highlighted by surrounded rectangles in gray. **b** PCA of SAFE scores of EMP metadata and taxa. The top 10 metadata identified by *tmap* are highlighted (markers with edge color of gray) and annotated with their names. Marker size is scaled according to SAFE enriched score. Colors of metadata are the same as that in the ranking, and taxa are in red. **c** Co-enrichment network of EMPO classes. Node colors are based on EMPO classes. Edge width of the network is the same as that of Fig. 5. Interconnections among the nodes in the network reflect the hierarchy of EMPO levels. Child classes of higher levels are connected to their parent classes of lower levels and are interconnected to each other. **d** Co-enrichment network of host metadata (host scientific name). Classification of the hosts are curated manually and colored accordingly. The co-enrichment network indicates that hosts of the same class appear to be more co-enriched when accounting for their association with the Earth’s microbiomes

In addition to the human gut microbiome datasets analyzed above, we also applied *tmap* to the large-scale microbiome samples from the Earth Microbiome Project (EMP) to extract their ecological patterns [5]. The original study elucidated a multiscale pattern of microbiome diversity of different environment types. In addition, via combination of well-established microbial ecology knowledge [49, 50] and the observed microbial diversity of the EMP samples, the EMP Ontology (EMPO) was proposed for the classification of environmental microbiome samples [5]. Our re-analysis found that the SAFE enriched scores of metadata were consistent with their EMPO levels; that is, larger scores (reflecting bigger enrichment subnetworks) correspond to lower levels, and vice versa (Fig. 7a, b). As expected, classes of EMPO level-1 were ranked at the top, followed by classes of EMPO level-2, and then by EMPO level-3 (Fig. 7a). We also found that some of the Environment Ontology (ENVO) descriptors had SAFE enriched scores comparable to that of EMPO classes (Fig. 7b). For instance, the ENVO level-1 descriptors of terrestrial and aquatic biome were ranked among the top, close to EMPO level-1 classes. But these ENVO descriptors were associated with different subnetworks to that of EMPO (Additional file 9: Figure S9), indicating that they can classify environmental microbiomes in a different way. Furthermore, geographical covariates, such as elevation and latitude, were ranked among the classes of EMPO level-2 or level-3, suggesting their roles in characterizing environment types. It is also worth noting that technical indicators, such as extraction center, sequence length, and platform, were among classes of EMPO level-2 and therefore needed to be considered as strong confounding factors in the meta-analysis of the EMP samples (Fig. 7a, b).

In addition, *tmap* was used to construct a co-enrichment network of the EMPO metadata to reveal their interrelations. As shown in Fig. 7c, the EMPO classes were interconnected in a network whose configuration reflects their hierarchy levels, with nodes of lower level to have more connections with other nodes in the network. A further analysis of the host-associated samples revealed finer interrelations between hosts and their microbiome stratification, which could complement the EMPO system with more detailed classifications (Fig. 7d, Additional file 10: Figure S10). We also tried to identify driver taxa of different environment types by including both metadata and taxa in a TDA network. The result showed that distinct groups of microbial taxa were enriched with different EMPO level-1 classes (Fig. 7c, Additional file 9: Figure S9). Taken together, applying *tmap* to the large-scale EMP dataset enabled us to effectively extract the hidden multiscale ecological patterns and interrelations of environment types associated with the earth microbiome and to identify their detailed stratification for finer classifications.

## Discussion and conclusions

*tmap* is an integrative method for analyzing population-scale microbiome variation, allowing both stratification of complex microbiome landscape and association of metadata of hosts or environmental types. This method is powered by the *Mapper* algorithm [31] for topological data analysis, which has been shown effective in summarizing large-scale high-dimensional datasets and in capturing complex patterns. In contrast to other widely used linear regression-based methods, including *envfit*, *adonis*, and ANOSIM, *tmap* is capable of identifying complex nonlinear patterns in both synthetic and real microbiome datasets, allowed by the employed subnetwork enrichment analysis. Furthermore, the subnetwork enrichment analysis enables *tmap* to calculate the SAFE scores for systematically mapping all host metadata onto the underlying microbiome variation to extract their associations and interrelations. The potential of *tmap* in population-scale microbiome studies was extensively demonstrated in our re-analysis of three published datasets [5–7], i.e., nonlinear trends and subpopulation enrichments of microbial taxa identified in the FGFP dataset, microbiome stratifications associated with countries and lifestyles revealed in the AGP dataset, and associations of microbiomes with earth environment types in different scales illustrated in the EMP dataset. These results indicated that *tmap* is capable of bringing more specific and comprehensive insights to the microbiome datasets with the related population metadata.

To achieve a better performance of *tmap* on a given microbiome dataset, we provide clustering and topological covering parameters that can be tuned, along with optimization functions for their choice (http://tmap.readthedocs.io). As the *Mapper* algorithm employs density-based clustering and discards unclustered samples, *tmap* would perform better with a larger dataset that includes more data points from a microbiome landscape to construct a more faithful topological network representation. Another important aspect of the algorithm is the choice of the filter functions, which depends on the nature of the studied dataset and research questions to be addressed. In practice, dimension reduction methods are the most commonly used filters [30]. For microbiome data, one such method is PCoA, which has helped reveal many biological insights, such as microbiome variations among human body sites [51]. Major components of microbiome variation can be captured by the first two or three PCs of PCoA in microbiome studies [5–7], but there may still be a large quantity of variation remained in other PCs. For instance, there is a relatively large proportion of variance uncaptured by the first two PCs (67.91%, 87.10%, and 80.71% for the FGFP, AGP, and EMP datasets), reflecting the dimensional nature of these microbiome variations (Additional file 11: Figure S11). It is worth noting that *tmap* has an advantage of recovering the distance information from the original high-dimensional space that is not captured by the PCs used as filters, such as the first two PCs of PCoA (Additional file 12: Figure S12). As illustrated in Additional file 13: Figure S13, the clustering step of *tmap* uses the original distance to calculate and recover distinct clusters of samples in their original high-dimensional space, which otherwise might be superimposed on each other due to the loss of variation in the projection space. As a result, *tmap* is able to capture far more variation than that in the projection space by the PCs used as filters (*tmap* vs PCoA, regression *R*^*2*^ of 0.80 vs 0.51 for the FGFP dataset using PC1 and PC2 of PCoA, see the “Methods” section and Additional file 14: Figure S14 for more details). The recovered variance is also evident by comparing the projected distances between samples within a cover to their network distances constructed by *tmap* (*tmap* vs PCoA, CV of 1.90 vs 0.55 for the FGFP dataset, see Additional file 15: Figure S15 for other datasets).

In principle, *tmap* can use more than two PCs as filters, but increasing the number of filters (e.g., *n*) will lead to an exponential increase in the number of covers to be generated (e.g., *10*^*n*^ *covers* for a *resolution* of *10* on each filter), which then will result in too few samples to be clustered or a TDA network too sparse to have sufficient connectivity among nodes (see the “Methods” section for more details on network *sparseness*). As shown in Additional file 14: Figure S14, using the first four PCs as filters resulted in a TDA network with high sparseness (e.g., 82.88% for the FGFP dataset in PCoA). To ensure that there are enough and dense samples to be binned within each cover for clustering analysis and to obtain a TDA network with sufficient connection between nodes, we used only the first two PCs of PCoA (or two-dimensional projection in t-SNE or UMAP) as filters in *tmap*.

Although regression improvement can be achieved by projection using more PCs (or components to be projected), the performance of *tmap* in capturing the variation of the original high-dimensional space of microbiome samples appears robust to the number of PCs used as filters (*tmap* vs projection space, regression *R*^2^ of 0.64 ± 0.10 vs 0.46 ± 0.14, Additional file 14: Figure S14). Otherwise, the use of many PCs (such as four PCs) will lead *tmap* to generate a TDA network with few connections among nodes and therefore will compromise its ability to recover sufficient original microbiome variation (Additional file 14: Figure S14).

In the case that a set of target variables (e.g., a subset of metadata or microbiome features, rather than the overall microbiome variation) are of interest, we propose a supervised strategy to help choose the most suitable PCs to be used as filters. This strategy is based on the *proportion of total variance explained* by each PC [52], multiplied by the *enriched SAFE scores* of the target variables, to rank and select a subset of PCs as filters for final TDA network construction (see the “Methods” section and Additional file 16: Figure S16 for details). Applying this strategy to the FGFP dataset showed that the first two PCs (PC1 and PC2) actually gave the largest aggregated scores when all the metadata were taken as target variables (42.39% of the cumulative aggregated score). Moreover, given different target variables of interest, different PCs may be selected as the most informative filters (e.g., PC 8, 3 for age and PC 1, 2 for BSS in the FGFP dataset).

In addition to PCoA, which is widely used in microbiome analysis [13], other dimension reduction methods can also be used as filters, especially nonlinear and large-scale methods, such as t-SNE [29] and UMAP [27]. To examine the robustness of different dimension reduction methods as filters in *tmap* to detect host-microbiome associations, we applied PCoA, t-SNE, and UMAP to the FGFP dataset and compared their results with that of *envfit*. All three methods shared a significant common subset of host covariates in their top 10’s (4 in top 10, permutation test *p* value = 1e^−4^, see the “Methods” section for details, Additional file 17: Figure S17). Furthermore, all three methods consistently supported the observed differences in the effect size of association for the four host covariates as identified in the comparison between *tmap* and *envfit* (Additional file 17: Figure S17). In future research, one may expect that it is possible to combine the results from different parameters and filters, to construct an integrative TDA network for multiscale mapping [53] and for large collections of microbiome datasets.

Subnetwork enrichment analysis of the SAFE algorithm allows *tmap* to transform the values of a target variable into a vector of SAFE scores, corresponding to each node in a TDA network. Therefore, the association patterns of target variables can be compared quantitatively by their vectors of SAFE scores, using ordination or co-enrichment analysis (Fig. 1). For each target variable, a network-level association (designated *SAFE enriched score*) can be obtained by filtering and summing its SAFE scores of individual nodes (see the “Methods” for details). Like the *R*-squared in linear regression, the SAFE enriched score can be used as an effect size to compare between different host covariates for their associations with microbiome variation. It is worth noting, however, that the SAFE score is different from the correlation coefficient in linear regression in two aspects. First, it is able to detect subtle and complex associations, both linear and nonlinear, as demonstrated in our analysis of synthetic and real-world datasets. Second, SAFE scores can form a vector of values, representing all local subnetwork associations, which can be subjected to further analysis of the interrelationships between metadata. In contrast, the correlation coefficient in linear regression is only a value of correlation, which cannot be used to analyze interrelations between the association patterns of metadata, as we have done with SAFE scores.

Furthermore, SAFE scores allow us to use co-enrichment analysis to scrutinize whether interrelations between target variables represent confounding effects or biological associations with microbiome variations. For instance, a significant co-enrichment between a host covariate (such as *Gender*) and a taxon (such as *Roseburia*) may represent the outcome of host-microbiome interactions. Instead, a co-enrichment between a medication (such as β-lactam antibiotic) and a disease (such as chronic fatigue syndrome) is likely due to a confounding effect. Therefore, although SAFE scores are calculated independently (via independent random shuffle) for each metadata or microbiome features, co-enrichment analysis would capture both biologically meaningful intercorrelations and confounding effects. Interpretation of these interrelations should be based on our knowledge of host-microbiome interactions and the background of studies, as we have demonstrated in the analysis of the FGFP, AGP, and EMP datasets.

In conclusion, *tmap* is an integrative framework for analyzing population-scale microbiome variations and their association with hosts or environments. Based on topological data analysis, it is able to capture complex microbiome variations from high-dimensional datasets and recover the lost variation in their low-dimensional projection or embedding. Moreover, TDA network representation and subnetwork enrichment analysis endows *tmap* with the ability to extract complex host-microbiome association patterns, especially nonlinear associations that are hard to detect with currently available methods. In microbiome research, given our inadequate knowledge of the dynamics and complexity of host-microbiome interactions, especially at population-scale, innovative data-driven methods for discovering complex patterns of host-microbiome association are urgently needed [54]. In this regard, *tmap* could provide insights from both microbiome stratification and association analysis to inform further hypothesis-driven microbiome studies. *tmap* is provided as a software freely available at https://github.com/GPZ-Bioinfo/tmap, along with detailed tutorials and online documents (https://tmap.readthedocs.io).

## Methods

### *Mapper* algorithm for microbiome data analysis

*tmap* is based on the *Mapper* algorithm [31] for topological data analysis (TDA) to transform high-dimensional microbiome profiles of individual samples into a network representation that captures both local and global topological patterns from the profiles (Fig. 2a). This algorithm begins with projection of high-dimensional data points (representing microbiome profiles) into a low-dimensional space using *filter* functions. Usually, dimension reduction methods are used as filters to generate coordinates of data points in a low-dimensional space [30]. For instance, when PCA is used for dimension reduction, either one or two principal component(s) can be used as filter. If PC1 is used as filter, the coordinates of the points along PC1 will be generated. If both PC1 and PC2 are used as filter, the coordinates of the points in a two-dimensional space will be generated. Other functions may also be used as filter, such as the eccentricity or density of a dataset, or even a subset of the original dimensions, as long as they can generate coordinates of data points in a low-dimensional space.

After projection of the data points into a low-dimensional space, the *covering* step of *Mapper* partitions the space into a number of overlapping *covers* with equal size. The purpose of covering is to use covers of the low-dimensional space to capture its topological properties, i.e., a cover represents a local neighborhood of the projected data points. Therefore, a collection of covers represents all neighborhood information of the projected data points. To connect neighborhoods that are close to each other, overlaps between covers are retained in the covering step of the algorithm. As a result, covers and their overlaps capture both local neighborhood of points and their global interconnections.

Information of original distances between data points might be lost after dimension reduction or projection. For example, two points that are far apart in the high-dimensional space might be projected as close neighbors in the low-dimensional space. The *clustering* step of *Mapper* is performed to retain the distance information of the high-dimensional space. For each cover, points binned within it are clustered into different clusters based on their distances in the original space rather than their projected distances. For instance, after applying PCoA to microbiome profiles, all the subsets of samples that fall within each cover (a two-dimensional cover if the first two coordinates are used, such as PC1 and PC2) will be clustered independently. As a result, samples within a cover would be clustered into different clusters if they are quite different in their original microbiome profiles, although they might be close to each other in the projection space of PC1 and PC2.

The last step of *Mapper* is to generate a TDA network, in which a *node* represents a group of samples that are clustered together and a *link* occurs between two nodes if they share common samples in their clusters. The TDA network provides a compressive representation of high-dimensional microbiome profiles for exploring microbiome variations and for stratification and association analysis.

### SAFE algorithm and SAFE score

Once a TDA network of microbiome profiles is constructed, the values of metadata or microbiome features can be individually mapped to the network (Fig. 2b). For a given metadata, e.g., *age*, this mapping assigns a numerical attribute (called *mapping score*) to each node, by calculating its averaged values among samples in the node (e.g., mean *age*). We assume that a target variable non-randomly distributed among the TDA network indicates that its association with the underlying microbiome profiles is significant. Intuitively, if the nodes with high mapping scores are neighbors, interconnected to each other in the network, a pattern of subnetwork enrichment of these nodes can be observed. The significance level of the observed pattern can be calculated by permuting the mapping scores along the whole network. A non-random enrichment pattern will have significantly higher scores for the subnetwork compared to the randomly permuted scores.

To implement the above idea, *tmap* adopts the spatial analysis of functional enrichment (SAFE) algorithm for network enrichment analysis [36]. The algorithm was developed as a systematic method for annotating biological network and examining their functional association. We modified the original algorithm to calculate an enrichment score (designated *SAFE score*) for each node in a TDA network for a given target variable (metadata or microbiome features), as described in the following steps (and as depicted in Fig. 2b):

1. This algorithm starts with a TDA network and a target variable of metadata (e.g., *age*) or taxa. For each node *u* in the network, SAFE defines a local neighborhood of *u* by identifying any other nodes that are closer than or equal to a maximum distance threshold (*d*) to *u*. Node distance is measured as the shortest path length between nodes**.** By default, the maximum distance threshold *d* is set to be equal to the *0.5th* percentile of all pairwise node distances in the network.
2. For each node, SAFE sums the values of neighbor nodes for a target variable as an observed neighborhood score (*S*_observed_). Meanwhile, permuted neighborhood scores (*S*_permuted_) are obtained by randomly shuffling the target variable among nodes in the network. The enrichment significance of the observed neighborhood score (*P*) is measured as the probability that a random score will fall between the observed neighborhood score (*S*_observed_) and the largest value of all scores (via ranking of both observed and permuted scores, as illustrated in Fig. 2b). Finally, the enrichment significance (*P*) is transformed into an enrichment score (*O*), designated as SAFE score, which is normalized in a range from 0 to 1 as below:
  $$ {O}_u=\frac{-{\log}_{10}\left(\max \left({P}_u,\frac{1}{n+1}\right)\right)}{-{\log}_{10}\left(\frac{1}{n+1}\right)} $$

where *n* is the number of shuffles, *P*_*u*_ is the significance of enrichment of node *u*, and *O*_*u*_ is the SAFE score of node *u*. Random shuffle is performed independently for each target variable.

3) A node is considered to be significantly enriched under a cutoff *p* value of 0.05 (which can be tuned in *tmap*). This cutoff *p* value can be translated to a cutoff SAFE score as below:

$$ {O}_{\mathrm{cutoff}}=\frac{-{\log}_{10}0.05}{-{\log}_{10}\left(\frac{1}{n+1}\right)} $$

Under the above cutoff value (*O*_cutoff_), *SAFE enriched score* is defined as the sum of SAFE scores of all significantly enriched nodes in a TDA network to measure the overall enrichment significance in the whole network, which can be used to filter or rank metadata or taxa.

### Microbiome datasets and sample metadata

The FGFP, AGP, and EMP microbiomes were collected from the data repositories provided in their publications, along with sample metadata (host phenotypes or environment types). We used the available OTU/sOTU tables from the original studies to avoid bias of 16S rRNA sequence data processing for result comparison [55]. The FGFP dataset comprises 1106 fecal samples and 69 identified host covariates, which were classified into seven metadata categories (anthropometric, lifestyle, blood parameters, health, bowel habit, dietary habit, and medication). The AGP dataset comprises 9496 fecal samples and 451 self-reported metadata. The EMP dataset comprises 2000 samples, and their metadata was downloaded from the EMP FTP site (see the “Availability of data and materials” section). The original OTU/sOTU tables were rarified and normalized to obtain an equal number of reads for each sample before further analysis. Beta-diversity (Bray-Curtis or unweighted UniFrac) distance matrix was calculated with *scikit-bio* (http://scikit-bio.org), followed by principal coordinates analysis (PCoA). For the AGP and EMP dataset, in which representative sequences were available, we re-annotated their taxonomy using the *usearch sintax* software (using a cutoff bootstrap value of 0.8) [56, 57]. We used genus-level profiles for the analysis in this study. To perform network enrichment analysis with *tmap*, categorical metadata was transformed into one-hot encoding with *scikit-learn*. Only metadata that is collected for at least 90% of samples was retained for further analysis. Missing values were filled with medians for all the retained metadata.

### Parameters for topological data analysis and network enrichment analysis

We used the same beta-diversity as the original studies (Bray-Curtis distance matrix for FGFP, unweighted UniFrac distance matrix for AGP and EMP) for result comparison. *tmap* used the first two principal coordinates of PCoA as lenses (filters). Different topological and clustering parameters were chosen for the datasets depending on their sample size and microbiome variation (FGFP: *overlap* = 0.75, *resolution* = 40, and *percentile eps threshold* = 95th; AGP: *overlap* = 0.95, *resolution* = 120, and *percentile eps threshold* = 90th; EMP: *overlap* = 0.75, *resolution* = 45, and *percentile eps threshold* = 95th). An online guide is available on how to choose proper parameters for a given dataset (see the “Availability of data and materials” section). After obtaining a TDA network for a dataset, metadata or taxon abundance was individually mapped to the network by enrichment analysis, using the SAFE algorithm. *p* values were calculated for the observed SAFE scores for each node in the TDA network by permutation test (*iterations* = 5000) and were FDR corrected for all the nodes. Nodes with a *p* value of ≥ 0.05 (FDR corrected) were considered significantly enriched and were used to calculate the SAFE enriched score for metadata or taxa.

### Identifying and ranking microbiome-associated covariates

For the FGFP dataset, ranking of the target variables of microbiome-associated host covariates was compared between *envfit*, *adonis*, ANOSIM, and *tmap*. Ten thousand permutations were used in *envfit*, *adonis*, and ANOSIM. Effect sizes were used to rank the covariates by these methods (*R*-squared of *envfit* and *adonis*, *R* value of ANOSIM and SAFE enriched score of *tmap*). Kendall’s tau test was used to statistically compare the rankings of *envfit* and *tmap*. Significant *p* value can be obtained for a consistent ranking of a subset of covariates, which are examined in a stepwise test from top to bottom. Results of the first two stepwise test were absent because Kendall’s tau test is valid only for a ranking with more than two covariates.

### Synthetic microbiomes and simulation of associations between microbiome and metadata

Synthetic microbiome datasets were generated with *SparseDOSSA*, using a Bayesian hierarchical log-normal distribution model to simulate species abundances [37]. Model parameters are estimated by fitting to a reference microbiome dataset. Four microbiome datasets, including the default template dataset of *SparseDOSSA*, FGFP dataset, AGP dataset, and EMP dataset, were used to train the model independently and the best one was chosen to further simulate associations of metadata (Additional file 1: Figure S1). Associations between metadata and microbiome were simulated by mapping values of metadata onto the PCoA spaces of microbiome variation (PC1 and PC2, using Bray-Curtis distance matrix) via various functions. Both linear and nonlinear associations were simulated with the corresponding mapping functions as follows.

Linear associations were generated by the following function:

$$ f\left( PC1, PC2\right)=a\times PC1+b\times PC2 $$

where the coefficients *a* and *b* are randomly chosen from the range of [− 1, 1] for each metadata; PC1 and PC2 are the coordinates of a microbiome sample in the two-dimensional PCoA space.

Nonlinear associations of multiple local enrichments were simulated by mapping Gaussian mixtures onto the PCoA space, using the following function:

$$ f\left( PC1, PC2,n\right)=\frac{1}{n}\sum \limits_{i=1}^n\exp \left(-\left[\frac{{\left( PC1-{\mu}_{i1}\right)}^2}{2{\sigma}^2}+\frac{{\left( PC2-{\mu}_{i2}\right)}^2}{2{\sigma}^2}\right]\right) $$

where *n* (that is 2 or 3 in our simulation) is the number of Gaussians to be simulated in the mixture; (*μ*_*i*1_, *μ*_*i*2_) is the center of the *i*th Gaussian in the PCoA space, and *σ is* the standard deviation; PC1 and PC2 are the coordinates of a microbiome sample in the two-dimensional PCoA space.

In order to use ANOSIM for microbiome association analysis, we also simulated categorical variables with linear or nonlinear patterns of associations. Binary discretization of continuous variables (with simulated linear associations as described above) was performed to obtain categorical variables. Based on the median of continuous variable, data points (samples in a PCoA space, PC1 and PC2) were assigned to two categorical groups (labeled as “True” if larger than the median, labeled as “False” otherwise, Additional file 18: Figure S18). For the simulation of categorical variables with nonlinear associations, we used an approach similar to the above simulation of multiple local enrichments. Instead of Gaussian mixtures, this approach picks multiple circular areas from the PCoA space and assigns samples within the areas as “True” and other samples as “False.” First, a number of random samples were selected from the PCoA space to be used as centers. For each categorical variable, this number is randomly chosen in the range from 1 to 5. Second, for each area, the 50 samples that are closest to its center (including the center itself) were included, according to their Euclidean distances on the PCoA space. If a selected sample is already included in other circular areas, it will be skipped and the next closest one is considered. Therefore, the ratio of sample sizes between the two categorical groups (“True” or “False”) was kept in the range from 1:9 to 1:1, given that there were a total of 500 samples in our simulation. As in the case of continuous variables, a mixed simulation comprises both linear and nonlinear associations, in a ratio of 1:3 in their numbers of categorical variables.

We used the default template microbiome dataset and model parameters of *SparseDOSSA* to generate synthetic microbiomes consisted of 500 samples. Three scenarios were designed to compare the performance of *tmap* and other methods in detecting associated metadata, including scenarios of linear associations only, nonlinear associations only, and the mix of both of them. In the first two scenarios, 50 associated metadata were generated according to the above mapping functions as positive cases to be detected; 50 random shuffles of the generated metadata were used as negative cases. In the mixed scenarios, four kinds of associations (200 in total, 50 for each kind) were generated and mixed, including Gaussian mixture with three symmetric centers, Gaussian mixture with three asymmetric centers, Gaussian mixture with two centers, and linear distribution as described above. At the same time, random shuffles of the generated metadata were used as negative cases. Performance in detecting positive cases of associated metadata was compared between *tmap* (measured by SAFE enriched score) and other methods (measured by *p* value) via receiver operating characteristic curve (ROC) and area under the curve (AUC) score. Significant difference between AUC scores (100 repeats of simulations) was accessed by Mann-Whitney *U* test. More details and the codes for the simulation can be found in the online Jupyter notebook (see the “Availability of data and materials” section).

### Stratification, enterotype, and ordination analysis with SAFE scores

Taxa-driven stratification of the TDA network of microbiome variation was obtained by identifying the most significant enriched genus (with the highest SAFE score among all genera) for each node in the network. A cutoff value of SAFE score ≥ 0.35 (corresponding to a negative log-transformed *p* value of 0.05 with 5000 iterations by the SAFE algorithm) was used to filter out nodes with no significant genus. The stratification was visualized by coloring the TDA network according to the enriched genera, which resulted in enterotype-like clusters in the microbiome landscape. Each cluster was highlighted by a color specific to its enriched genus. For comparison, traditional enterotype analysis was also performed using the partitioning around medoids (PAM) method (Jensen-Shannon divergence, and a preset number of three clusters) [23]. Ordination of the SAFE scores of metadata and taxa was done by PCA, to visualize how they relate to each other after mapping to the microbiome variation. Metadata or taxa that share similar enrichment subnetworks will be close to each other within the PCA space.

### Co-enrichment network analysis

SAFE scores of metadata or taxa contain information about their co-enrichment patterns on a TDA network, which can be used to calculate their interrelations when accounting for their association with microbiome variation. First, for each feature (metadata or taxa), we separated all the nodes into two groups: one group of enriched nodes (as defined in the above SAFE algorithm) and another group of the remaining nodes. Therefore, for each pair of features, a contingency table can be obtained based on the combination of their node groups. Next, Fisher’s exact test was used to examine the independence of node groups, and its *p* value was used for co-enrichment network construction. Only positive dependence of node groups was considered as a co-enrichment relationship. The resulted network was filtered using a threshold of *0.5th* percentile of the *p* values (FDR corrected). The negative log-transformed *p* value of the test was used as edge weight for each pair of co-enriched features in a co-enrichment network.

### Selection and evaluation of filter functions in *tmap* for microbiome data analysis

We used PCoA for the projection of the FGFP microbiome profiles to a low-dimensional space and demonstrated a strategy on the selection and evaluation of PCs as filters in *tmap* given a chosen set of target variables of interest. First, each individual PC was used as the only filter in *tmap* to construct a TDA network. After that, SAFE enriched scores of the chosen target variables were calculated based on the network and were summed to quantify the overall association of all the variables. The summed score for each PC was then multiplied by *the ratio of variance explained* of the PC to calculate an *aggregated score*. At last, all the PCs were ranked according to their aggregated scores. Accumulation curve of the ranked aggregated scores was then used to determine how many and which PCs to be selected as filters in a final analysis given a specified threshold value (see Additional file 16: Figure S16 for a workflow of the steps). The specified threshold value determines how much of the cumulative aggregated scores to be kept by the selected PCs (e.g., 70%).

Different dimension reduction methods, including PCoA, t-SNE, and UMAP, were compared to evaluate the robustness of *tmap*. In the comparison, two components were selected from each method to be used as filters in *tmap*. And all the methods used the same set of parameters (*overlap* = 0.75, *resolution* = 40, and *percentile eps threshold* = 95th). To assess the significance of the observed number of common covariates in the top 10’s of the rankings from each method, permutation test is used. In detail, three pseudo-rankings were obtained by permuting the 69 covariates three times independently for each iteration to calculate a random number of common covariates in the top 10’s from the pseudo-rankings. After 10,000 iteration, the observed value was compared with the random values to obtain its rank (*r*) in a descending order, and *p* value was calculated as *r*/10000.

### Recovering complex microbiome variations from high-dimensional space

*tmap* is able to recover the original complex microbiome variations that are lost in a low-dimensional projection space, such as in the PCoA space of PC1 and PC2 (Additional file 13: Figure S13). Linear regression analysis was performed to quantify the variation captured by *tmap* than that in the low-dimensional projection after using filters of dimension reduction methods. *R*-squared (*R*^*2*^) was obtained from the linear regression between the *original distance* in the original high-dimensional microbiome profiles and the *projected distance* in the projection space, or the *network distance* in *tmap*, respectively. In this study, the original distance is the Bray-Curtis distance between samples in their original high-dimensional microbiome profiles. The projected distance is the Euclidean distance between samples in the projection space. Network distance was measured as the minimal number of edges to be traversed (or shortest path) between each pair of nodes. Because network distances were calculated between nodes, in which nodes are groups of samples, the corresponding original distances between samples from two nodes (*u*, *v*) are calculated as below:

$$ {d}_{\mathrm{original}}=\frac{1}{nm}\sum \limits_i^n\sum \limits_j^md\left({u}_i,{v}_j\right) $$

where *n* and *m* are the number of samples in node *u* and *v* respectively; *u*_*i*_ is the *i*th sample in node *u*, and *v*_*j*_ the *j*th sample in node *v*; and *d* (*u*_*i*_, *v*_*j*_) is the Bray-Curtis distance between sample *u*_*i*_ and *v*_*j*_.

In this study, we defined a *sparseness* metric of a TDA network to quantify the overall connectivity among its nodes as below:

$$ \mathrm{Sparseness}=1-\frac{2\times \left|\left\{\left(u,v\right)\ \right|\ u\ \mathrm{and}\ v\ \mathrm{are}\ \mathrm{connected}\Big\}\right|}{n\left(n+1\right)} $$

where *n* is the total number of nodes in the TDA network and *u* and *v* are two nodes in the network. Self-connections (e.g., *u* = *v*) are also counted. The greater the value of the sparseness of a TDA network indicates that the larger number of node pairs that are not connected and therefore cannot be measured by the network distance.

Comparison between low-dimensional projection and *tmap* using the above regression analysis was performed for different dimension reduction methods (including PCoA, PCA, t-SNE, UMAP) and also for different number of components used as filters (from top two to four components). We also compared samples that are binned within a same cover in the projection space to measure the difference in variance captured by different methods; that is, distances between samples or nodes from different covers were not included in the comparison. The obtained network distances and projected distances (from within each cover) were normalized into the range of [0, 1], by dividing the distance to the maximum distance from the overall network or the overall projection space respectively. Coefficient of variation (CV), the ratio of the standard deviation to the mean, was calculated for both the network distances and the projected distances to compare the variance captured by each of the methods (*tmap* vs dimension reduction) when constrained within individual covers.

## Supplementary information

**Additional file 1: Figure S1.** Comparison between simulated data and training data for different microbiome datasets via principal coordinate analysis (PCoA). Bray-Curtis distance matrix is used in the analysis. PCoA plots show the similarity between simulated data and training data of (a) FGFP, (b) EMP, (c) AGP and (d) the demo data of *SparseDOSSA*, respectively.

**Additional file 2: Figure S2.** Performance of *tmap* in detecting linear and nonlinear patterns of simulated microbiome associations for different number of metadata. Receiver operating characteristic (ROC) curves are used to compare the performance between (a) *tmap* and * adonis*, (b) *tmap* and ANOSIM, (c) *tmap* and *envfit* , in detecting microbiome-associated metadata. Categorical metadata are used for the comparison between *tmap* and ANOSIM. Continuous metadata are used in other cases. Three scenarios of association with different number of metadata are examined (including linear-only, nonlinear-only and a mix of both). The shaded areas indicate 95% confidence intervals (100 repeats). (d) One-sided (greater) t-test is used to test the significance of improved area under the curve (AUC) scores of *tmap* over the other three methods (*envfit*, *adonis* and ANOSIM respectively).

**Additional file 3: Figure S3.** Illustrations of *tmap* in the detection of associations of simulated metadata**.** Color legend (from blue to red) indicates values of metadata (from small to large). Network color represents SAFE scores on each node.

**Additional file 4: Figure S4.** Comparison of rankings of host covariates associated with the FGFP microbiomes using *envfit*, *adonis*, ANOSIM and *tmap*.

**Additional file 5: Figure S5.** Example of large variances of a host covariate in a local subnetwork that lead to low SAFE scores. Left, PCoA plot of samples colored according to the host covariate of time since previous relief. Right, TDA network colored according to the SAFE scores of time since previous relief. The zoomed area shows a local subnetwork with a large variance of the covariate, which results in low SAFE scores. Node colors are based on their SAFE scores, from red (large values) to blue (small values).

**Additional file 6: Figure S6.** Illustrations of TDA network enrichment analysis of metadata compared with PCoA. (a,c,e) PCoA plots of microbiome samples of the FGFP cohort, colored according to the covariates of Gender:F, Time since previous relief and HDL cholesterol, respectively. (b,d,f) TDA network enrichment scores (SAFE scores) of the covariates of Gender:F, Time since previous relief and HDL cholesterol, respectively. Colors are based on their values, from red (large values) to blue (small values).

**Additional file 7: Figure S7.** In-depth stratification of the FGFP microbiomes. (a) Stratification based on traditional enterotype analysis. Nodes are colored based on enterotype driver taxa. (b) Stratification based on the most enriched taxon, which is identified from all taxa by comparing their SAFE scores on each node. Node colors are based on the identified taxon. Only taxa enriched in more than 100 nodes are highlighted. Remaining unstratified nodes (with no enriched taxa) are colored in gray.

**Additional file 8: Figure S8.** PCA of the SAFE scores of taxa and host covariates shows the overall pattern of their associations with the AGP microbiomes. The top 10 covariates and taxa identified by SAFE enriched scores are highlighted (markers with edge color of gray) and annotated with their names. Host covariates are colored based on metadata category, and taxa are in red. Marker size is scaled according to the SAFE enriched score of metadata or taxa.

**Additional file 9: Figure S9.** Comparison of TDA network enrichment patterns between classes of EMPO level-1 and ENVO_biome level-1. Enriched subnetworks of the EMP microbiomes are identified and colored based on the classes of EMPO level-1 (a) and classes of ENVO_biome level-1 (b), respectively. Only enriched nodes are colored and showed in the network. The remaining nodes are colored in gray.

**Additional file 10: Figure S10.** TDA network enrichment patterns of host-associated microbiomes. Nodes in the TDA network of the EMP microbiomes are colored based on their enriched host. Classification of the hosts are curated manually. Only enriched nodes are colored and showed in the network. The remaining nodes are colored in gray.

**Additional file 11: Figure S11.** Proportion of total variance explained by each PC in PCoA of the FGFP, AGP and EMP datasets.

**Additional file 12: Figure S12.** Regression between original distance and projected distance or network distance for the FGFP, AGP and EMP datasets respectively. PC1 and PC2 of PCoA are used as filters for *tmap* analysis.

**Additional file 13: Figure S13.** Recovering microbiome variations from their original high dimensional space by *tmap*. After the projection of high dimensional microbiome profiles into a low dimensional space (such as PC1 and PC2 of PCoA), original variations might be lost. For example, two distinct clusters of samples (colored orange and blue) from the original space are superimposed on each other in the projection space. The *clustering* step of *tmap* performs clustering analysis using their original distances to recover the separated clusters from the original space. The recovered variation is captured by *tmap* in its network representation.

**Additional file 14: Figure S14.** Better regression performance of *tmap* in capturing microbiome variations in high dimensional space than dimension reduction methods. Each panel shows the linear regressions between the original distance and the projected distance (at the left), or the network distance (at the right), for different dimension reduction methods (panels along the row), and for different number of components used as filters (panels along the column). R-squared (*R*^*2*^) is shown for each regression. The proportion of pairs of nodes that are not connected is indicated by the TDA network *sparseness* metric.

**Additional file 15: Figure S15.** More variance captured by TDA network distance in *tmap* than the projected distance for samples within a same cover. For each pair of nodes constructed from the same cover, projected distances (colored blue) and network distances (colored red), both normalized into the range of [0, 1], are plotted against their original distances. Coefficient of variation (CV) is shown to indicate the variances captured for different datasets: (a) the AGP dataset, (b) the EMP dataset, (c) the FGFP dataset.

**Additional file 16: Figure S16.** Workflow of evaluation and selection of PCs as filters in *tmap* for a subset of target variables. The workflow begins with principal coordinates analysis (PCoA) of a microbiome dataset to obtain individual PCs and the proportion of total variance explained by each PC. *Aggregated scores* of the chosen target variables are then calculated for each PC by using the PC as filter in *tmap*. Ranking and accumulation curve of the aggregated scores is then employed to select the most suitable PCs for a final *tmap* analysis, according to a specified threshold of the cumulative aggregated scores. Details of score calculation are shown alongside each step.

**Additional file 17: Figure S17.** Comparison of different reduction methods as filters in *tmap*. Bristol stool scale (BSS), time since previous relief, mean corpuscular hemoglobin (MCH) and shift work are indicated in red. The four common host covariates in the top 10’s of the rankings from different methods are shown in bold text.

**Additional file 18: Figure S18.** Illustrations of simulated nonlinear associations for categorical metadata.

**Additional file 19: Text S1.** Descriptions of the simulations of circular and spiral association patterns.

**Additional file 20: Table S1.** Comparison of the performances in detecting simulated metadata between *envfit*, *adonis*, ANOSIM and *tmap*.

**Additional file 21: Table S2**. Detection of host covariates significantly associated with the FGFP microbiomes using *envfit*, *adonis*, ANOSIM and *tmap*.

**Additional file 22: Table S3.** Co-enrichment subnetworks of pet past 3 months and its co-enriched features of the FGFP microbiomes.

**Additional file 23: Table S4.** Ordination results of the SAFE scores of metadata and taxa for the datasets of FGFP, AGP and EMP.

**Additional file 24: Table S5.** Comparison of the stratification of the AGP microbiomes between *tmap* and PAM based clustering.

**Additional file 25:** Review history.

## Data Availability

GitHub source of *tmap* is available under the GNU General Public License v3.0 at https://github.com/GPZ-Bioinfo/tmap [58]. Online documents of *tmap*: https://tmap.readthedocs.io/. The version of the source code of *tmap* during the current study is available at 10.5281/zenodo.3228979 [59]. Jupyter notebooks of the source codes for data analysis, simulated datasets, microbiome datasets, and sample metadata: https://github.com/GPZ-Bioinfo/tmap_notebook. Datasets used in this study were obtained from the following public sources: FGFP dataset [7] from ENA (https://www.ebi.ac.uk/ega/studies/EGAS00001001689) [60], AGP dataset [6] from ENA (https://www.ebi.ac.uk/ena/data/view/PRJEB11419) [61], and EMP dataset [5] from Earth Microbiome Project (ftp://ftp.microbio.me/emp/release1) [62].
