# Supplementary figures and images for: tmap: an integrative framework based on topological data analysis for population-scale microbiome stratification and association studies
Source: Genome Biol. 2019 Dec 23;20:293. doi: 10.1186/s13059-019-1871-4 (PMC6927166; doi:10.1186/s13059-019-1871-4)

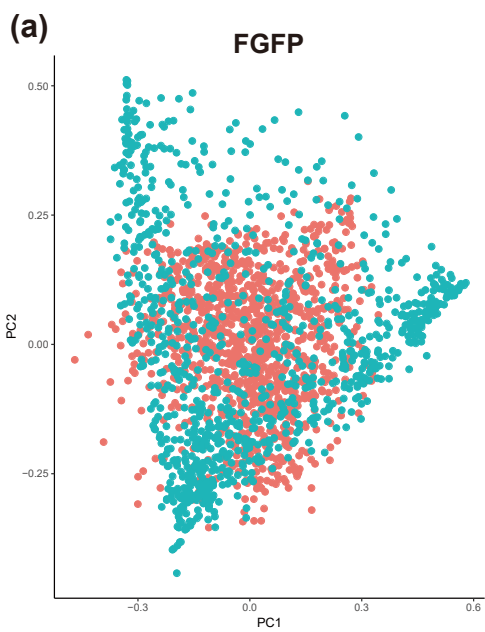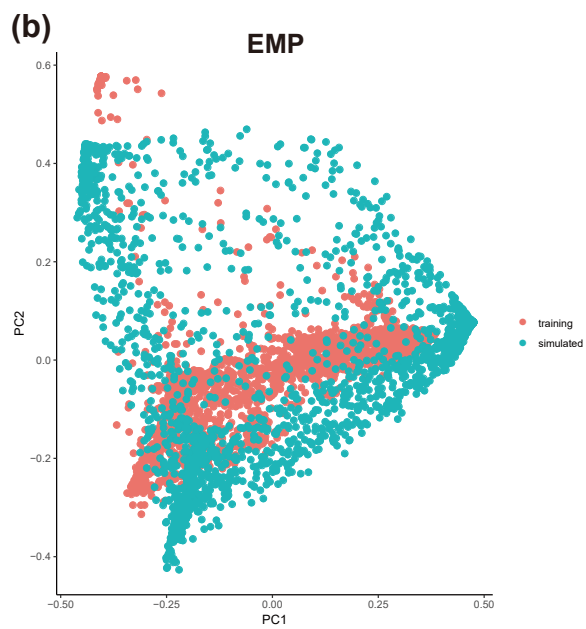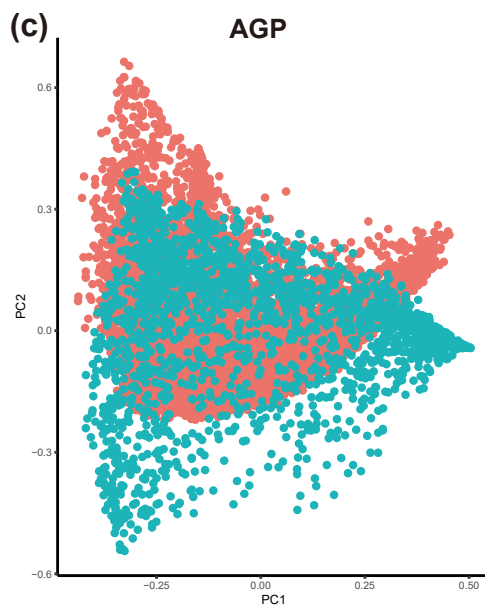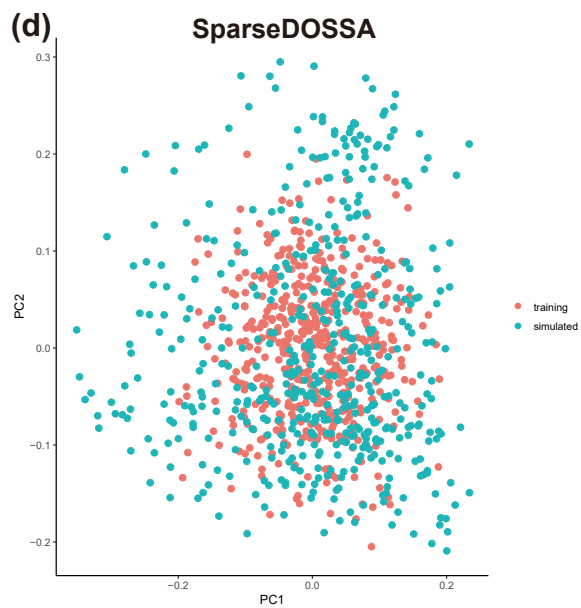

Supplement: Supplementary file 1 — Additional file 1: Figure S1. Comparison between simulated data and training data for different microbiome datasets via principal coordinate analysis (PCoA). Bray-Curtis distance matrix is used in the analysis. PCoA plots show the similarity between simulated data and training data of (a) FGFP, (b) EMP, (c) AGP and (d) the demo data of SparseDOSSA, respectively. [file 13059_2019_1871_MOESM1_ESM.pdf]

(a)

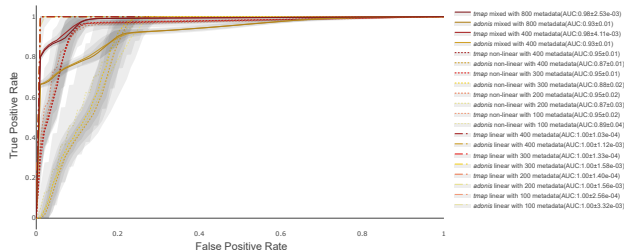

(b)

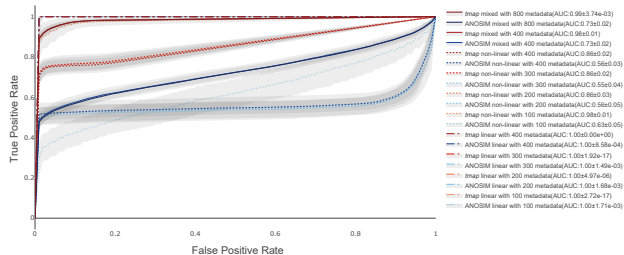

(c)

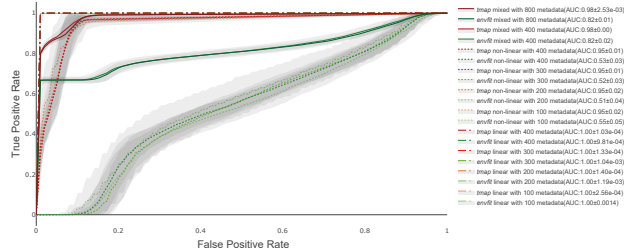

(d)

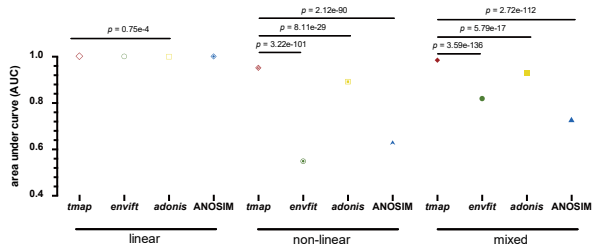

Supplement: Supplementary file 2 — Additional file 2: Figure S2. Performance of tmap in detecting linear and nonlinear patterns of simulated microbiome associations for different number of metadata. Receiver operating characteristic (ROC) curves are used to compare the performance between (a) tmap and adonis, (b) tmap and ANOSIM, (c) tmap and envfit , in detecting microbiome-associated metadata. Categorical metadata are used for the comparison between tmap and ANOSIM. Continuous metadata are used in other cases. Three scenarios of association with different number of metadata are examined (including linear-only, nonlinear-only and a mix of both). The shaded areas indicate 95% confidence intervals (100 repeats). (d) One-sided (greater) t-test is used to test the significance of improved area under the curve (AUC) scores of tmap over the other three methods (envfit, adonis and ANOSIM respectively). [file 13059_2019_1871_MOESM2_ESM.pdf]

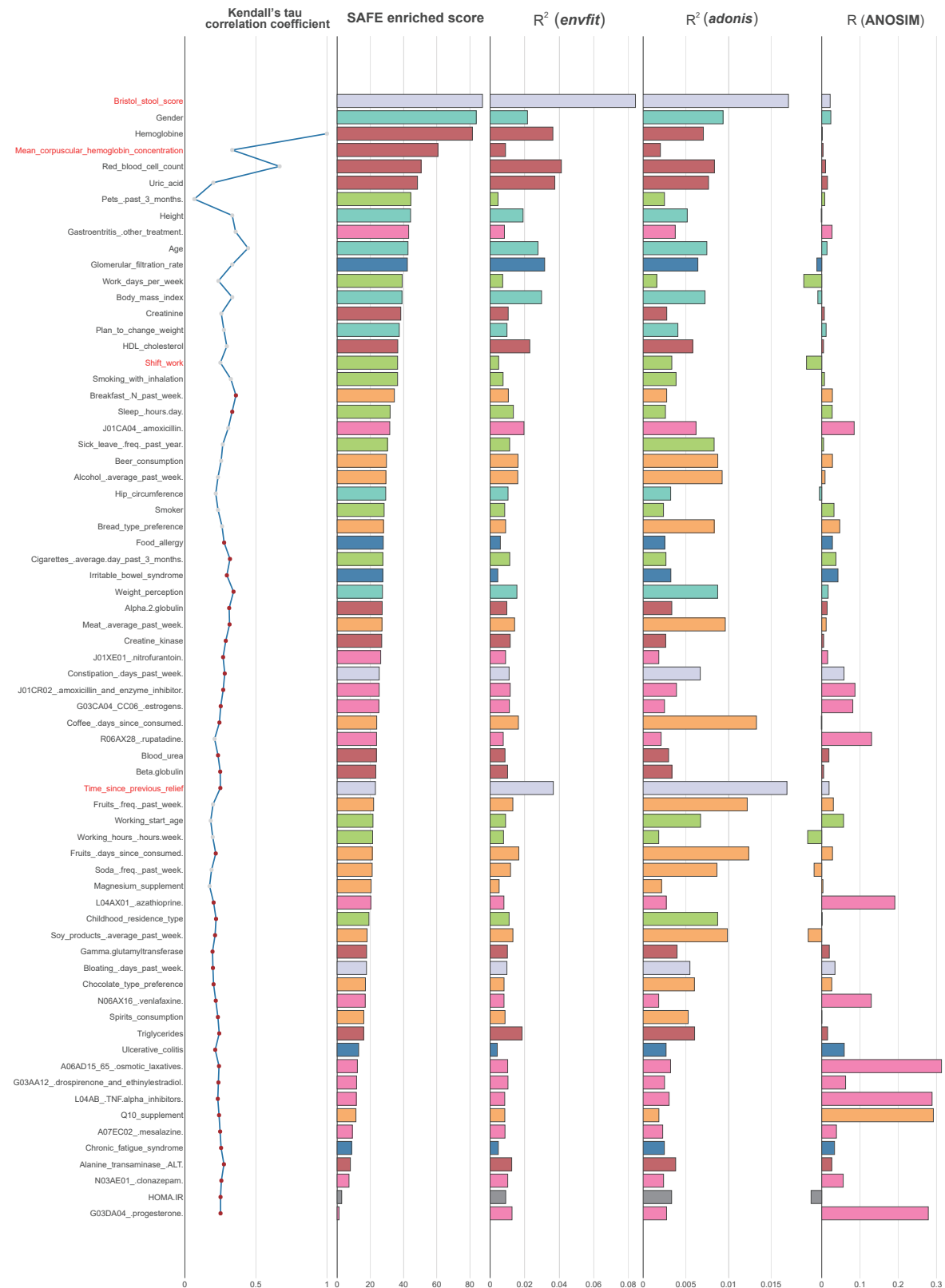

Supplement: Supplementary file 4 — Additional file 4: Figure S4. Comparison of rankings of host covariates associated with the FGFP microbiomes using envfit, adonis, ANOSIM and tmap. [file 13059_2019_1871_MOESM4_ESM.pdf]

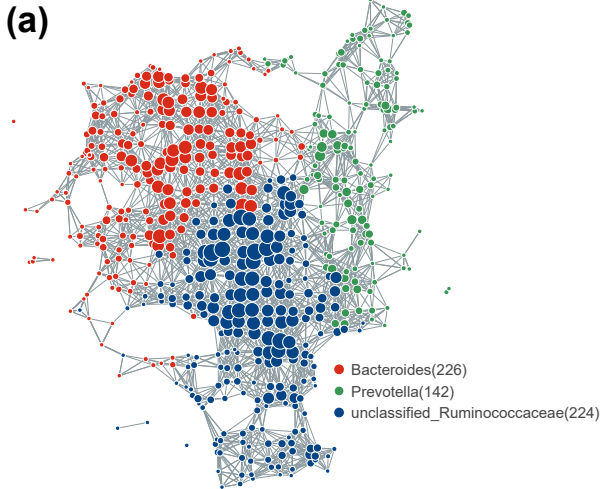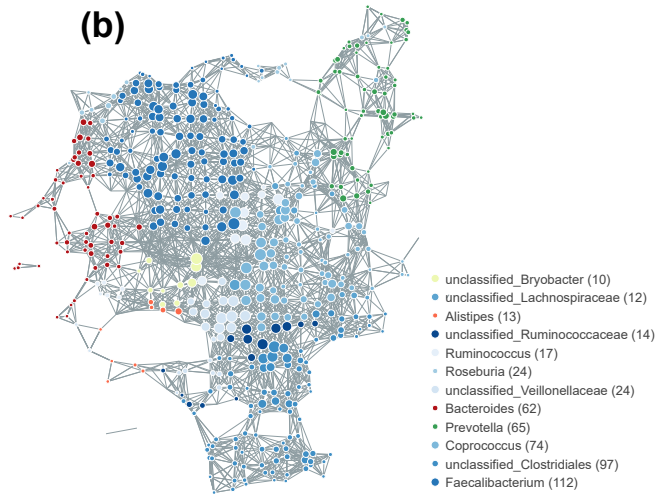

Supplement: Supplementary file 7 — Additional file 7: Figure S7. In-depth stratification of the FGFP microbiomes. (a) Stratification based on traditional enterotype analysis. Nodes are colored based on enterotype driver taxa. (b) Stratification based on the most enriched taxon, which is identified from all taxa by comparing their SAFE scores on each node. Node colors are based on the identified taxon. Only taxa enriched in more than 100 nodes are highlighted. Remaining unstratified nodes (with no enriched taxa) are colored in gray. [file 13059_2019_1871_MOESM7_ESM.pdf]

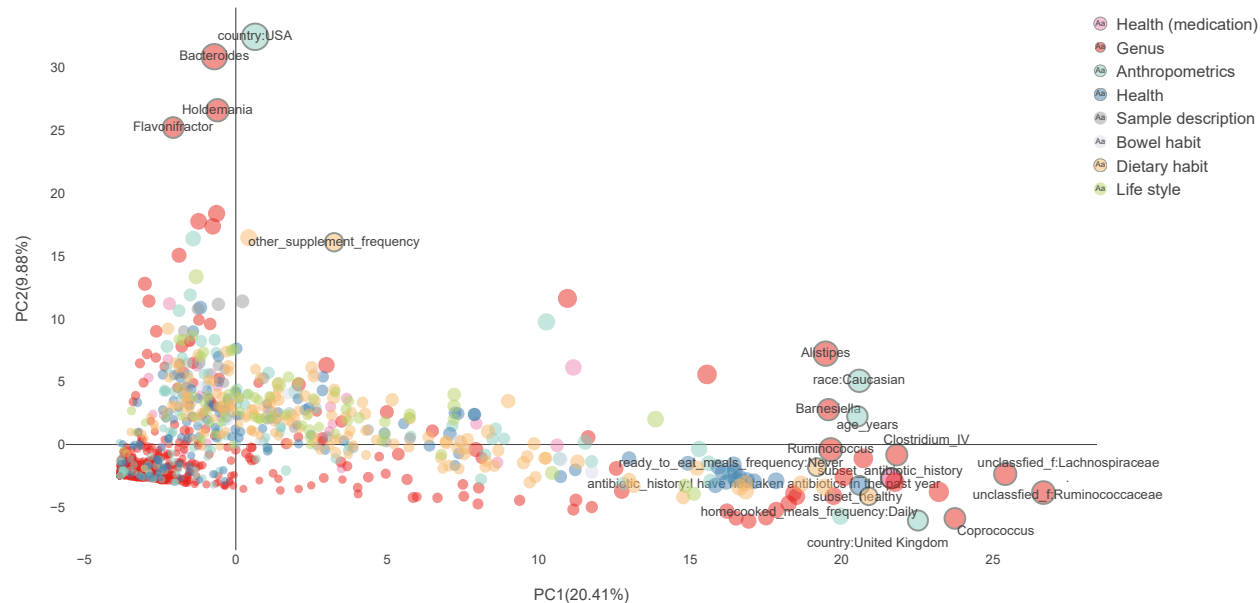

Supplement: Supplementary file 8 — Additional file 8: Figure S8. PCA of the SAFE scores of taxa and host covariates shows the overall pattern of their associations with the AGP microbiomes. The top 10 covariates and taxa identified by SAFE enriched scores are highlighted (markers with edge color of gray) and annotated with their names. Host covariates are colored based on metadata category, and taxa are in red. Marker size is scaled according to the SAFE enriched score of metadata or taxa. [file 13059_2019_1871_MOESM8_ESM.pdf]

**(a)**

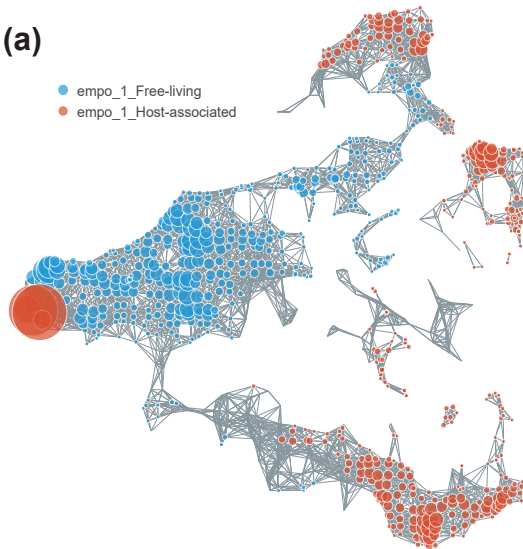

**(b)**

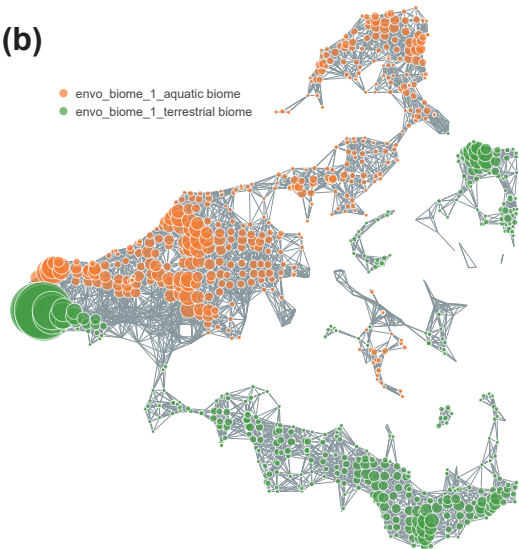

Supplement: Supplementary file 9 — Additional file 9: Figure S9. Comparison of TDA network enrichment patterns between classes of EMPO level-1 and ENVO_biome level-1. Enriched subnetworks of the EMP microbiomes are identified and colored based on the classes of EMPO level-1 (a) and classes of ENVO_biome level-1 (b), respectively. Only enriched nodes are colored and showed in the network. The remaining nodes are colored in gray. [file 13059_2019_1871_MOESM9_ESM.pdf]

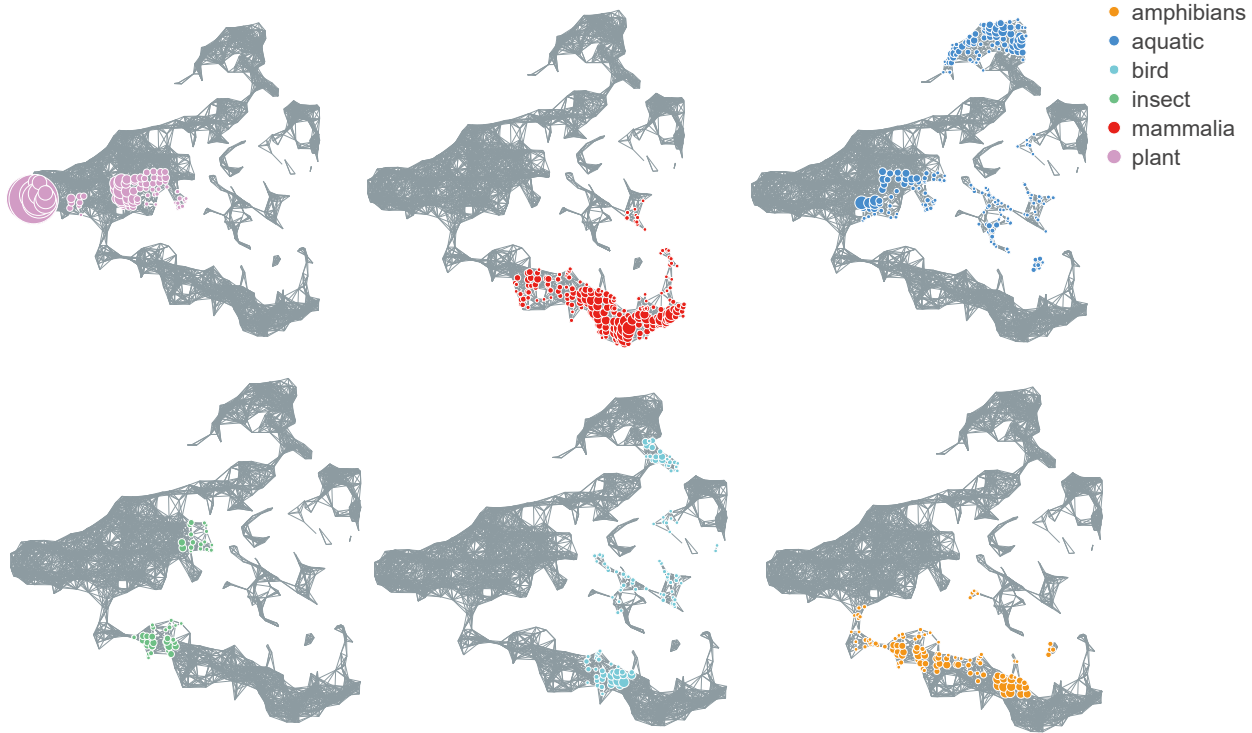

Supplement: Supplementary file 10 — Additional file 10: Figure S10. TDA network enrichment patterns of host-associated microbiomes. Nodes in the TDA network of the EMP microbiomes are colored based on their enriched host. Classification of the hosts are curated manually. Only enriched nodes are colored and showed in the network. The remaining nodes are colored in gray. [file 13059_2019_1871_MOESM10_ESM.pdf]

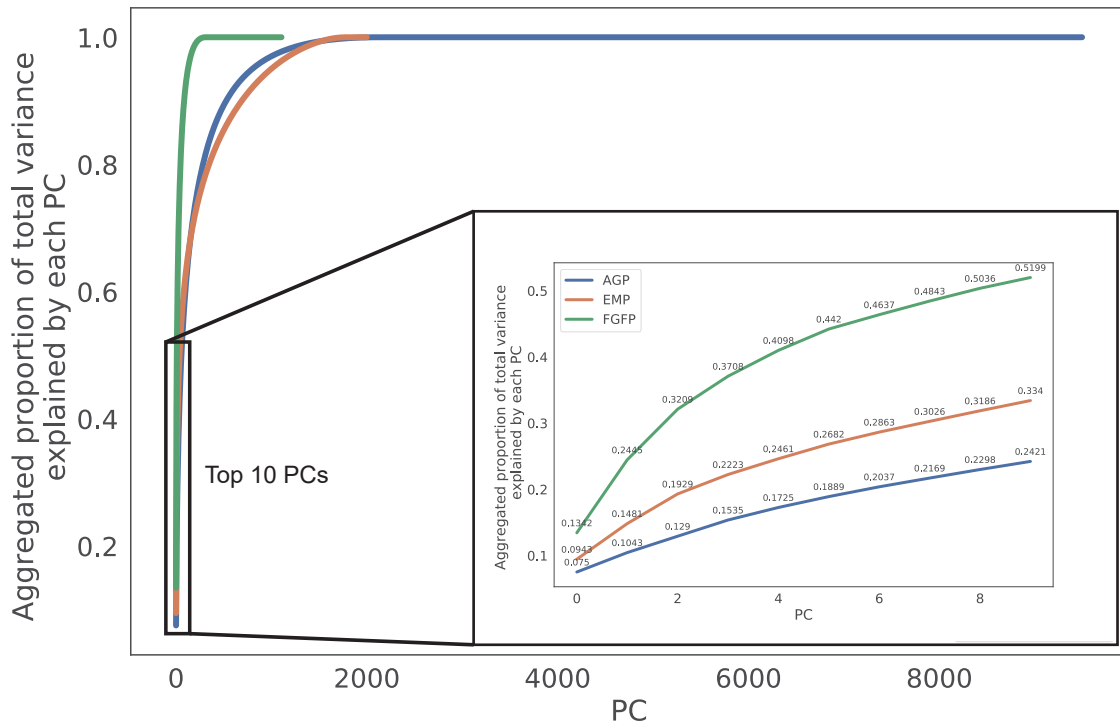

Supplement: Supplementary file 11 — Additional file 11: Figure S11. Proportion of total variance explained by each PC in PCoA of the FGFP, AGP and EMP datasets. [file 13059_2019_1871_MOESM11_ESM.pdf]

# Recover high dimensional information *via* the *clustering* step of *tmap*

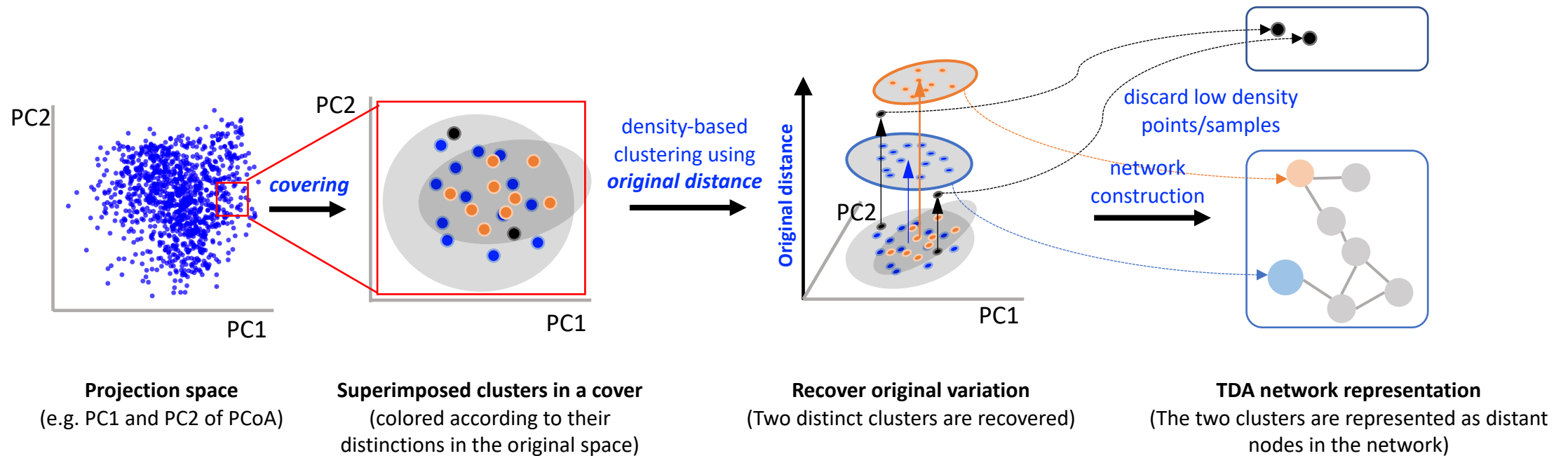

Supplement: Supplementary file 13 — Additional file 13: Figure S13. Recovering microbiome variations from their original high dimensional space by tmap. After the projection of high dimensional microbiome profiles into a low dimensional space (such as PC1 and PC2 of PCoA), original variations might be lost. For example, two distinct clusters of samples (colored orange and blue) from the original space are superimposed on each other in the projection space. The clustering step of tmap performs clustering analysis using their original distances to recover the separated clusters from the original space. The recovered variation is captured by tmap in its network representation. [file 13059_2019_1871_MOESM13_ESM.pdf]

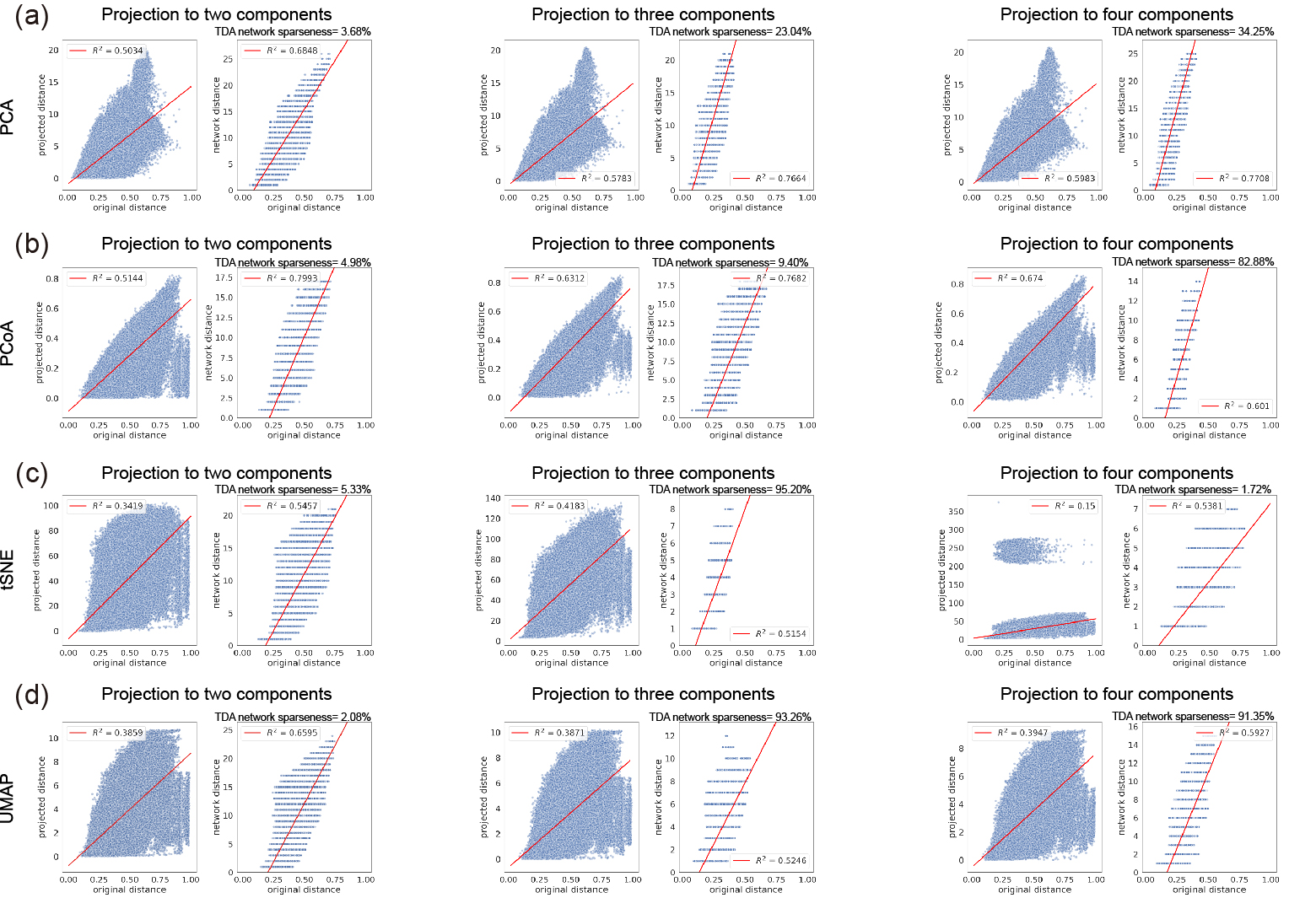

Supplement: Supplementary file 14 — Additional file 14: Figure S14. Better regression performance of tmap in capturing microbiome variations in high dimensional space than dimension reduction methods. Each panel shows the linear regressions between the original distance and the projected distance (at the left), or the network distance (at the right), for different dimension reduction methods (panels along the row), and for different number of components used as filters (panels along the column). R-squared (R2) is shown for each regression. The proportion of pairs of nodes that are not connected is indicated by the TDA network sparseness metric. [file 13059_2019_1871_MOESM14_ESM.jpg]

(a)

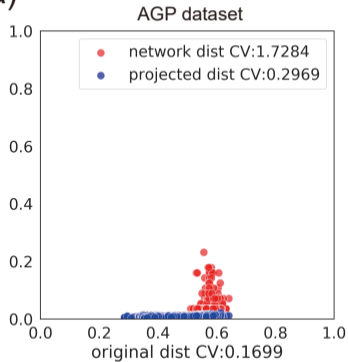

(b)

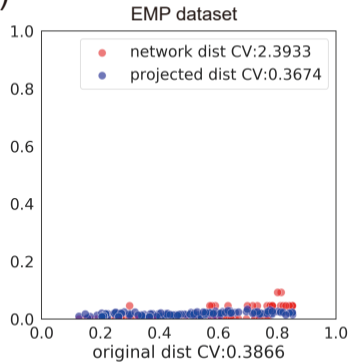

(c)

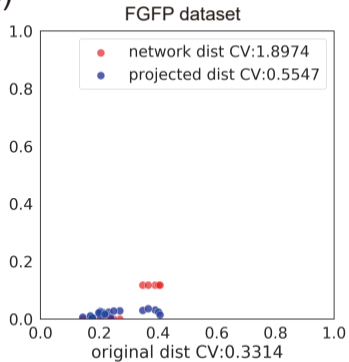

Supplement: Supplementary file 15 — Additional file 15: Figure S15. More variance captured by TDA network distance in tmap than the projected distance for samples within a same cover. For each pair of nodes constructed from the same cover, projected distances (colored blue) and network distances (colored red), both normalized into the range of [0, 1], are plotted against their original distances. Coefficient of variation (CV) is shown to indicate the variances captured for different datasets: (a) the AGP dataset, (b) the EMP dataset, (c) the FGFP dataset. [file 13059_2019_1871_MOESM15_ESM.pdf]

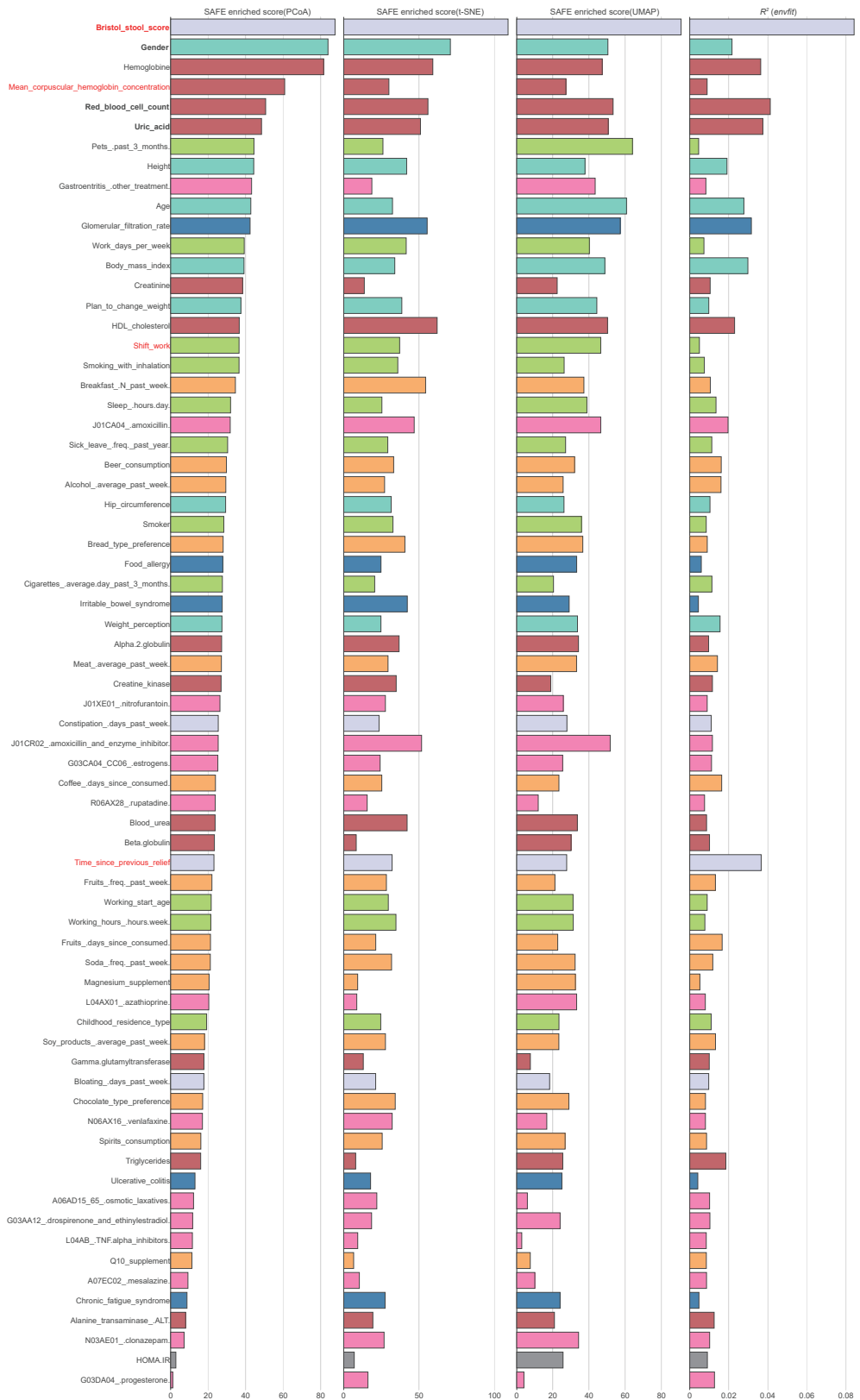

Supplement: Supplementary file 17 — Additional file 17: Figure S17. Comparison of different reduction methods as filters in tmap. Bristol stool scale (BSS), time since previous relief, mean corpuscular hemoglobin (MCH) and shift work are indicated in red. The four common host covariates in the top 10’s of the rankings from different methods are shown in bold text. [file 13059_2019_1871_MOESM17_ESM.pdf]

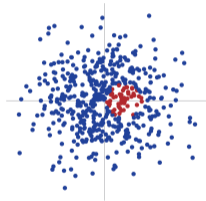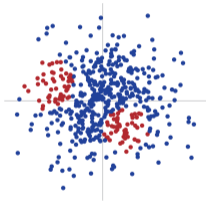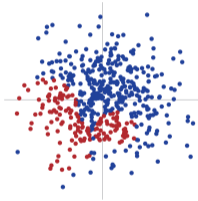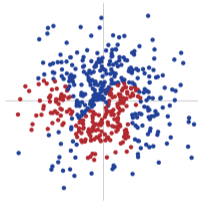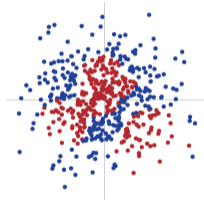

● False  
● True

Supplement: Supplementary file 18 — Additional file 18: Figure S18. Illustrations of simulated nonlinear associations for categorical metadata. [file 13059_2019_1871_MOESM18_ESM.pdf]
