# Supplementary material for: tmap: an integrative framework based on topological data analysis for population-scale microbiome stratification and association studies
Source: Genome Biol. 2019 Dec 23;20:293. doi: 10.1186/s13059-019-1871-4 (PMC6927166; doi:10.1186/s13059-019-1871-4)
Supplement: Supplementary file 3 — Additional file 3: Figure S3. Illustrations of tmap in the detection of associations of simulated metadata. Color legend (from blue to red) indicates values of metadata (from small to large). Network color represents SAFE scores on each node. [file 13059_2019_1871_MOESM3_ESM.pdf]

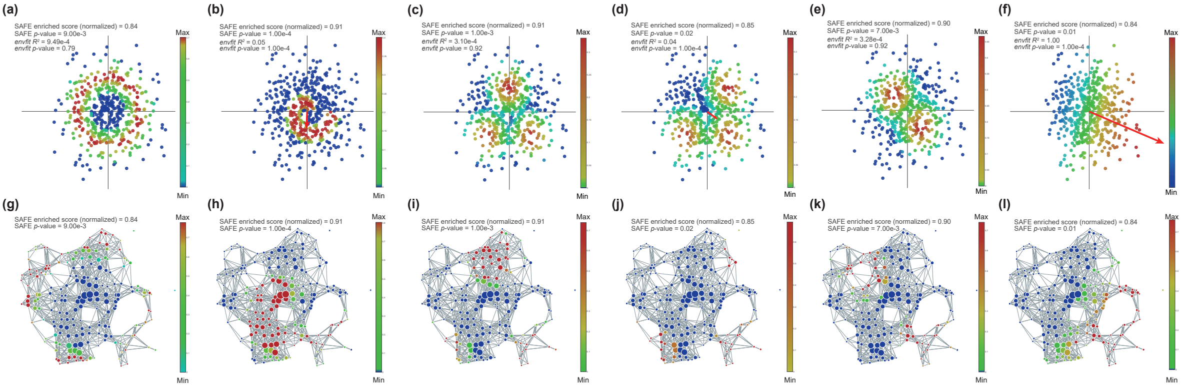
(a)
SAFE enriched score (normalized) = 0.84
SAFE p-value = 9.00e-3
envfit R² = 9.49e-4
envfit p-value = 0.79
(b)
SAFE enriched score (normalized) = 0.91
SAFE p-value = 1.00e-4
envfit R² = 0.05
envfit p-value = 1.00e-4
(c)
SAFE enriched score (normalized) = 0.91
SAFE p-value = 1.00e-3
envfit R² = 3.10e-4
envfit p-value = 0.92
(d)
SAFE enriched score (normalized) = 0.85
SAFE p-value = 0.02
envfit R² = 0.04
envfit p-value = 1.00e-4
(e)
SAFE enriched score (normalized) = 0.90
SAFE p-value = 7.00e-3
envfit R² = 3.28e-4
envfit p-value = 0.92
(f)
SAFE enriched score (normalized) = 0.84
SAFE p-value = 0.01
envfit R² = 1.00
envfit p-value = 1.00e-4
(g)
SAFE enriched score (normalized) = 0.84
SAFE p-value = 9.00e-3
(h)
SAFE enriched score (normalized) = 0.91
SAFE p-value = 1.00e-4
(i)
SAFE enriched score (normalized) = 0.91
SAFE p-value = 1.00e-3
(j)
SAFE enriched score (normalized) = 0.85
SAFE p-value = 0.02
(k)
SAFE enriched score (normalized) = 0.90
SAFE p-value = 7.00e-3
(l)
SAFE enriched score (normalized) = 0.84
SAFE p-value = 0.01
Max
Min
