# Supplementary material for: tmap: an integrative framework based on topological data analysis for population-scale microbiome stratification and association studies
Source: Genome Biol. 2019 Dec 23;20:293. doi: 10.1186/s13059-019-1871-4 (PMC6927166; doi:10.1186/s13059-019-1871-4)
Supplement: Supplementary file 5 — Additional file 5: Figure S5. Example of large variances of a host covariate in a local subnetwork that lead to low SAFE scores. Left, PCoA plot of samples colored according to the host covariate of time since previous relief. Right, TDA network colored according to the SAFE scores of time since previous relief. The zoomed area shows a local subnetwork with a large variance of the covariate, which results in low SAFE scores. Node colors are based on their SAFE scores, from red (large values) to blue (small values). [file 13059_2019_1871_MOESM5_ESM.pdf]

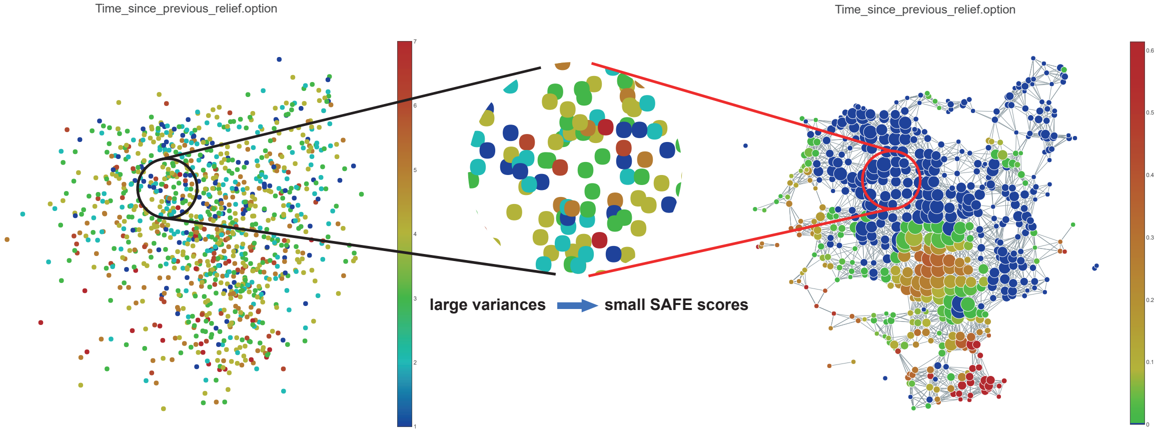

Time_since_previous_relief.option
7
6
5
4
3
2
1
large variances
small SAFE scores
Time_since_previous_relief.option
0.6
0.5
0.4
0.3
0.2
0.1
0
