# Supplementary material for: tmap: an integrative framework based on topological data analysis for population-scale microbiome stratification and association studies
Source: Genome Biol. 2019 Dec 23;20:293. doi: 10.1186/s13059-019-1871-4 (PMC6927166; doi:10.1186/s13059-019-1871-4)
Supplement: Supplementary file 6 — Additional file 6: Figure S6. Illustrations of TDA network enrichment analysis of metadata compared with PCoA. (a,c,e) PCoA plots of microbiome samples of the FGFP cohort, colored according to the covariates of Gender:F, Time since previous relief and HDL cholesterol, respectively. (b,d,f) TDA network enrichment scores (SAFE scores) of the covariates of Gender:F, Time since previous relief and HDL cholesterol, respectively. Colors are based on their values, from red (large values) to blue (small values). [file 13059_2019_1871_MOESM6_ESM.pdf]

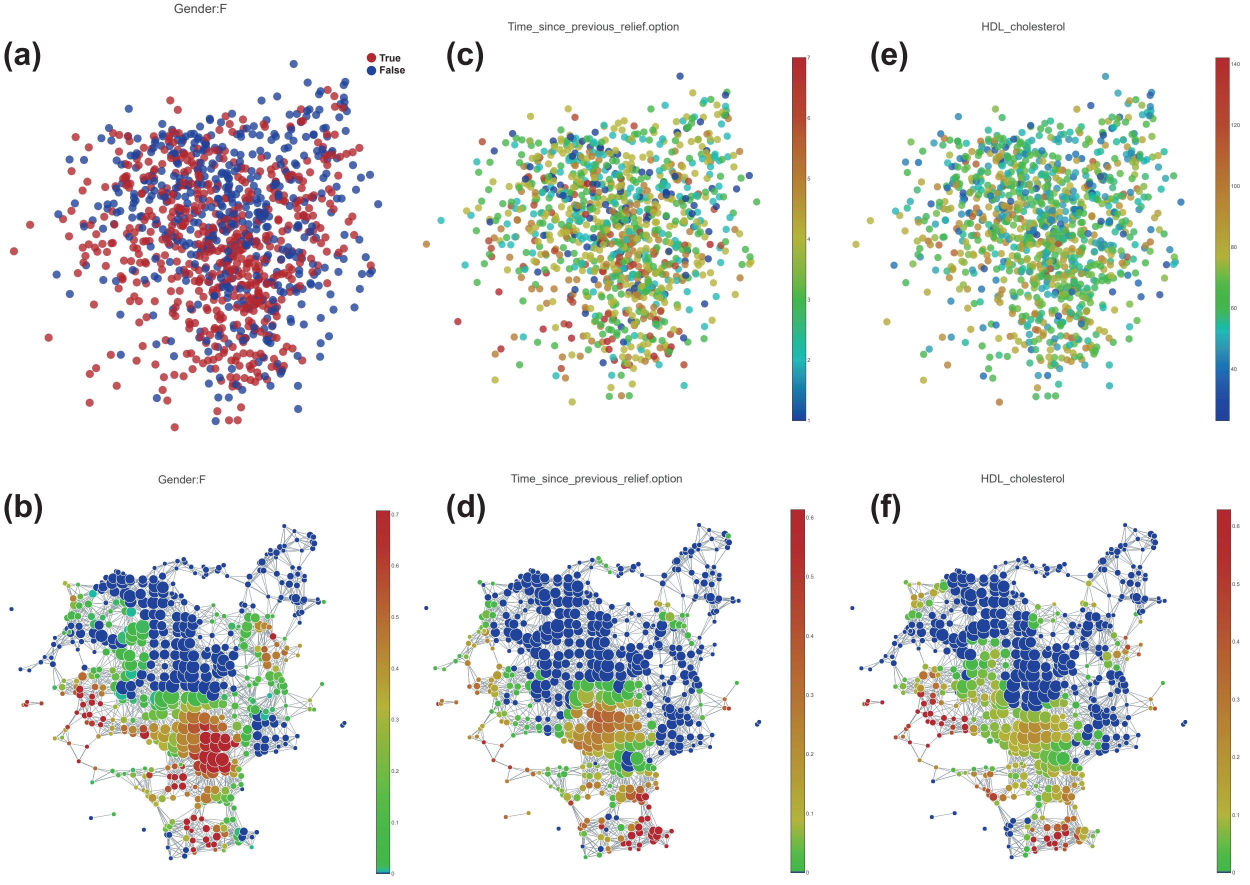

Gender:F
(a)
True
False
Time_since_previous_relief.option
(c)
1
2
3
4
5
6
7
HDL_cholesterol
(e)
40
60
80
100
120
140
Gender:F
(b)
0
0.1
0.2
0.3
0.4
0.5
0.6
0.7
Time_since_previous_relief.option
(d)
0
0.1
0.2
0.3
0.4
0.5
0.6
HDL_cholesterol
(f)
0
0.1
0.2
0.3
0.4
0.5
0.6
