# Supplementary material for: tmap: an integrative framework based on topological data analysis for population-scale microbiome stratification and association studies
Source: Genome Biol. 2019 Dec 23;20:293. doi: 10.1186/s13059-019-1871-4 (PMC6927166; doi:10.1186/s13059-019-1871-4)
Supplement: Supplementary file 12 — Additional file 12: Figure S12. Regression between original distance and projected distance or network distance for the FGFP, AGP and EMP datasets respectively. PC1 and PC2 of PCoA are used as filters for tmap analysis. [file 13059_2019_1871_MOESM12_ESM.pdf]

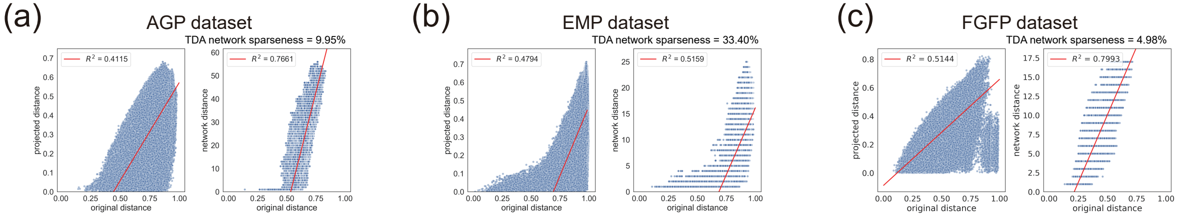
(a)
AGP dataset
TDA network sparseness = 9.95%
R^2 = 0.4115
projected distance
original distance
R^2 = 0.7661
network distance
original distance
(b)
EMP dataset
TDA network sparseness = 33.40%
R^2 = 0.4794
projected distance
original distance
R^2 = 0.5159
network distance
original distance
(c)
FGFP dataset
TDA network sparseness = 4.98%
R^2 = 0.5144
projected distance
original distance
R^2 = 0.7993
network distance
original distance
