# Supplementary material for: tmap: an integrative framework based on topological data analysis for population-scale microbiome stratification and association studies
Source: Genome Biol. 2019 Dec 23;20:293. doi: 10.1186/s13059-019-1871-4 (PMC6927166; doi:10.1186/s13059-019-1871-4)
Supplement: Supplementary file 16 — Additional file 16: Figure S16. Workflow of evaluation and selection of PCs as filters in tmap for a subset of target variables. The workflow begins with principal coordinates analysis (PCoA) of a microbiome dataset to obtain individual PCs and the proportion of total variance explained by each PC. Aggregated scores of the chosen target variables are then calculated for each PC by using the PC as filter in tmap. Ranking and accumulation curve of the aggregated scores is then employed to select the most suitable PCs for a final tmap analysis, according to a specified threshold of the cumulative aggregated scores. Details of score calculation are shown alongside each step. [file 13059_2019_1871_MOESM16_ESM.pdf]

Supervised selection of PCs based on target variables of metadata or microbiome features

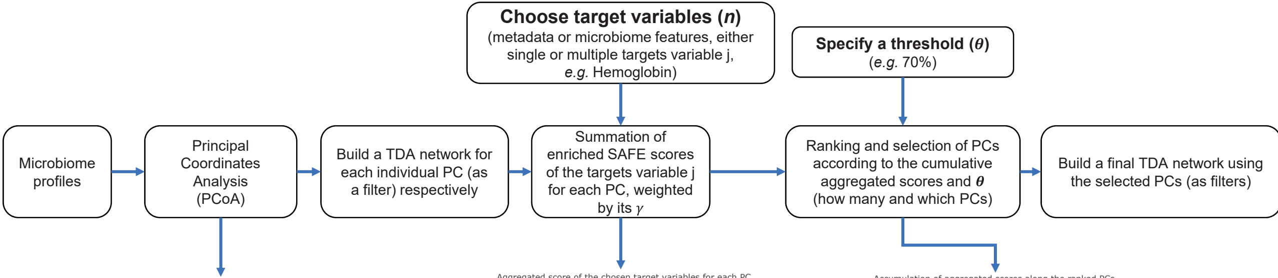

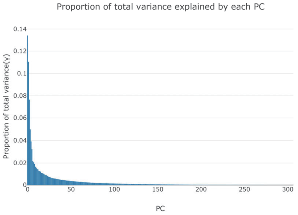

$\gamma_i = \frac{\lambda_i}{\sum_i^m \lambda_i}$

λ_i: eigenvalue of PC_i
m: number of PCs
γ_i: proportion of total variance explained by PC_i

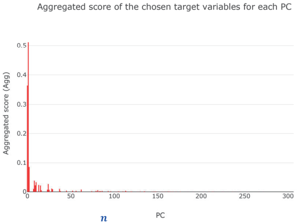

$Agg_i = \gamma_i \sum_j^n S_j$

γ_i: proportion of total variance explained by PC_i
S_j: enriched SAFE score for target variable j
n: number of target variables
Agg_i: aggregated score of the target variables for PC_i

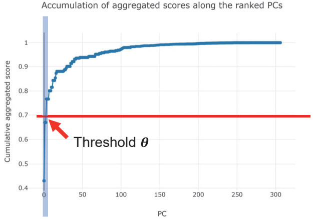

Selected PCs: PC1 and PC2
