# Supplementary material for: tmap: an integrative framework based on topological data analysis for population-scale microbiome stratification and association studies
Source: Genome Biol. 2019 Dec 23;20:293. doi: 10.1186/s13059-019-1871-4 (PMC6927166; doi:10.1186/s13059-019-1871-4)

**Simulating spiral enrichment patterns of metadata associated with microbiome**

Circular enrichment patterns of metadata associated with microbiome in a PCoA space were generated according to the following formula:

$$d=\sqrt{{PC}_{1}^{2}+{PC}_{2}^{2}}$$

$$f\left( {PC}_{1}, {PC}_{2} \right)=\left\{ \begin{aligned} 0, &\mathrm{if} \left| R-d \right|>r; \\ \sqrt{r^{2}-\left( R-d \right)^{2}}, & otherwise. \end{aligned} \right.$$

where *r* is the radius of the circular band (colored red in the following illustration) and *R* is the radius of the central circle of circular band (blue dash-line in the following illustration). In our simulation, the PCoA space was centered at (0, 0). *R* and *r* were set to 1.5 and 0.7 respectively.


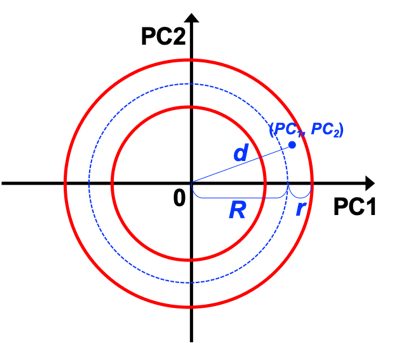


Spiral enrichment patterns of metadata associated with microbiome in a PCoA space were generated according to the following formula:

$$d=\sqrt{{PC}_{1}^{2}+{PC}_{2}^{2}}$$

$$f\left( {PC}_{1},{PC}_{2} \right)=\left\{ \begin{aligned} 0, &\mathrm{if} \left| R-d \right|>r; \\ \sqrt{r^{2}-{(R-d)}^{2}}, & otherwise. \end{aligned} \right.$$

$$R=\arctan(\frac{{PC}_{1}}{{PC}_{2}})+n*\pi, \left\{ \begin{aligned} n=0 ({PC}_{1}>0, {PC}_{2}>0) \\ n=1 ({PC}_{1}<0) \\ n=2 ({PC}_{1}<0, {PC}_{2}<0) \end{aligned} \right.$$

where *r* (*r* =1 in our simulation) is the radius of the spiral band (the light blue rectangle which moves along the red spiral curve in the following illustration). *R* is the radius of the spiral curve.


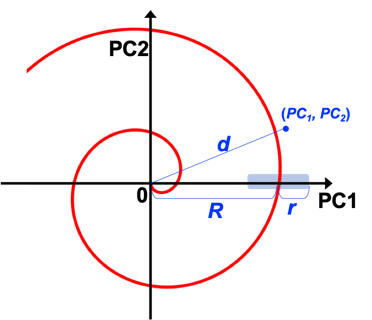

Supplement: Supplementary file 19 — Additional file 19: Text S1. Descriptions of the simulations of circular and spiral association patterns. [file 13059_2019_1871_MOESM19_ESM.docx]
