# Supplementary material for: tmap: an integrative framework based on topological data analysis for population-scale microbiome stratification and association studies
Source: Genome Biol. 2019 Dec 23;20:293. doi: 10.1186/s13059-019-1871-4 (PMC6927166; doi:10.1186/s13059-019-1871-4)
Supplement: Supplementary file 25 — Additional file 25: Review history. [file 13059_2019_1871_MOESM25_ESM.docx]

**1^st^ round**

**Reviewer 1**

As microbiome datasets are accumulating rapidly, analytical tools are badly needed to discover patterns hidden within those high dimensional datasets. Because existing tools are generally more capable to calculate linear patterns, it is necessary to develop new tools to uncover subtle and non-linear patterns. This paper developed a new analysis pipeline that utilized topological data analysis technique which is good at finding subtle and non-linear patterns within microbiome datasets. The author also demonstrated the advantages of their method, called tmap, by using simulated and real-world big data. Overall, I think their work is of high quality and priority and an important supplement to existing toolbox. Suggestions are as follows:

This manuscript requires their readers to be familiar with topological analysis, otherwise the readers might find it hard to understand its methodology. For example, what are covering and clustering in the second step of tmap? what is the purpose of these two operations and how they are done? How does topological analysis divide PCoA into several clusters? What is enrichment scores? It seems to be something comparable to correlation coefficient in linear regression? It would be great if the author gives more explanations to help their readers understand this method.

The author compared tmap and envfit to demonstrate the advantages of tmap over existing tools. It will be more credible if other existing tools, e.g., ADONIS, ANOSIM and etc., are also included in the comparisons.

Fig.2a,b,c. are they the same non-linear patterns? Especially Fig.2a and Fig.2b look very similar. What is the purpose of putting three figures of one pattern here? Is it possible to put different patterns here?

"However, there were differences in the ranking of several host covariates, including time since previous relief, mean corpuscular hemoglobin (MCH) and shift work (Fig. 3a). We speculate that the difference might be due to nonlinear association patterns that are captured by tmap but not by envfit.". This speculation will be supported if several (rather than only one) linear methods output the same rankings while tmap gives different rankings.

Fig.2d is not referenced in the manuscript.

**Reviewer 2**

Liao et al. developed a new algorithm for topological data analysis (TDA) for microbiome data. TDA is a powerful method to handle with complicated, highly dimensional dataset. Its application to the field of microbiome would be important. Authors have used synthetic data, the gut microbiome data from two different cohorts and the earth microbiome data to demonstrate its application. It sounds very promising. However, it is not easy to understand each step of the algorithms and the underlying rational. The fig 1, overview of the workflow does not provide sufficient information about underlying steps. A detailed algorithm workflow would be definitely needed to aid understanding.

The same problem is observed for the method part. It lacks sufficient information. To give you a few examples:

L28 on page 11: "filter/len function for mapping the original data points to their low dimentional coordinates, For instance, the first two principal coordinators of PCoA can be used as filters". What does "filters" mean? Are the first two sufficient? How to decide numbers of filters? What is the impact on the following steps? How robust it would be? Although authors said that detailed explanation and guidline are provided on line. It is still important to explain it clearly in the paper.

Page 12: Spatial analysis of functional enrichment (SAFE) algorithm for network enrichment analysis. The authors claimed that it can be applied to a target variable (or multiple target variables). It is unclear how it works for multiple target variables. The provided formula does not copy with multiple variables. L13 on page 13: "random shuffle is performed independently for each target variable when there are more than one". It is not a correct way, as target variables can be inter-correlated/confounding.

Page 8: enterotype-like stratification. The concept of enterotype has been abandoned in the field (see Knight et al. Cell Host Microbe. 2014 Oct 8;16(4):433-7). As shown in Fig 5, different groups can be formed based on the type of species under consideration. It is good to remove the concept of "enterotype".

L16: "a TDA network is constructed to represent the microbiome diversity". I don't understand it. How this network can represent the diversity?

**Response to reviewers**

**Reviewer #1**

This manuscript requires their readers to be familiar with topological analysis, otherwise the readers might find it hard to understand its methodology. For example, what are covering and clustering in the second step of tmap? what is the purpose of these two operations and how they are done?

Response: Thank you for the suggestions. We have now prepared a new figure (Fig. 7a) and updated the Methods part (line 11-27, page 14; line 1-15, page 15) of our revised manuscript to clearly explain the covering and clustering steps of tmap, together with descriptions of the purpose of these steps in microbiome data analysis. Algorithmic details are also intuitively illustrated in the new Fig. 7a. With these updates and the new Fig. 7a of the Mapper algorithm used within tmap, we believe that readers who are not familiar with topological data analysis can now easily understand our method and will find its potential in microbiome data analysis.

How does topological analysis divide PCoA into several clusters?

Response: Our revised manuscript now includes descriptions (line 7-11, page 15) to explain how clustering is performed to divide samples in a PCoA space into clusters, and their meanings in the context of microbiome data analysis.

What is enrichment scores? It seems to be something comparable to correlation coefficient in linear regression?

Response: We have revised our manuscript to clearly and intuitively explain the concept of enrichment score, designated as SAFE score in tmap (line 9-16, page 16; line 22-29, page 4; line 1-4, page 5), together with the rationale for using the scores in microbiome association analysis (line 18-26, page 15). A new figure (Fig. 7b) has been added to illustrate the algorithmic details on the calculation of the scores. And comparison with correlation coefficient in linear regression has also been discussed (line 8-21, page 13). We hope these updates will help our readers to better understand the scores and how the scores can be used to capture complex microbiome association patterns for further analysis.

It would be great if the author gives more explanations to help their readers understand this method.

Response: We appreciate this suggestion very much. We have revised our manuscript substantially to explain our method more intuitively, with more microbiome context, and with more succinct algorithm details. The updates and improvements are as follows:

1.We have revised Fig. 1 to present an overall workflow of tmap, focusing on the major steps and results of the pipeline;

2.We have added new figures to illustrate the Mapper and SAFE algorithm (Fig 7a,b), with key steps highlighted and annotated intuitively using schematic diagrams；

3.We have added thorough explanations and discussions for the Mapper algorithm and SAFE algorithm in the Methods (line 14-27, page 14; line 1-15, page 15; line 17-26, page 15) and Discussions (line 12-25, page 12; line 1-29, page 13) of our revised manuscript.

We believe that the tmap method has now been well explained for the readers unfamiliar with the concepts of topological data analysis and network enrichment analysis. Our revised manuscript now provides intuitive and solid technical background for the understanding and application of our method.

The author compared tmap and envfit to demonstrate the advantages of tmap over existing tools. It will be more credible if other existing tools, e.g., ADONIS, ANOSIM and etc., are also included in the comparisons.

Response: Thank you for this suggestion. We have now included ADONIS and ANOSIM in the comparison with tmap, using both synthetic dataset (revised Fig. 2; new supplementary Table S1) and the FGFP dataset (line 6-12, page 7; new supplementary Fig. S4), as we have done before for envfit. The new results of comparison support our previous claim of the advantages of tmap over linear methods. Accordingly, details on the simulation of categorical metadata for ANOSIM have been added in the Methods (line 8-23, page 19).

Fig.2a,b,c. are they the same non-linear patterns? Especially Fig.2a and Fig.2b look very similar. What is the purpose of putting three figures of one pattern here? Is it possible to put different patterns here?

Response: Our previous idea was to demonstrate multiple local enrichments as nonlinear microbiome associations in our simulation of metadata, and Fig 2a, b, c correspond to three different nonlinear patterns in terms of multiple local enrichments. Our purpose of including these nonlinear patterns in Fig. 2 was to present three possible scenarios of associations for more thorough comparisons between tmap the other linear methods: linear associations, nonlinear associations that cannot be detected with linear methods (Fig. 2a,c), and nonlinear associations that also can be detected with linear methods but weakly(Fig. 2b). Our revised manuscript now clearly explains this purpose (line 26-29, page 5; line 1-4, page 6; revised Fig. 2) According to the suggestion, we have also added a new supplementary figure S3 (new supplementary Text S1) to demonstrate other nonlinear patterns, including a circular pattern and a spiral pattern, along with the detection of these patterns by tmap and envfit.

"However, there were differences in the ranking of several host covariates, including time since previous relief, mean corpuscular hemoglobin (MCH) and shift work (Fig. 3a). We speculate that the difference might be due to nonlinear association patterns that are captured by tmap but not by envfit.". This speculation will be supported if several (rather than only one) linear methods output the same rankings while tmap gives different rankings.

Response: We have added two more methods (ADONIS and ANOSIM) in the analysis of host covariates. The results support the differences identified in our previous analysis. That is, the effect size of mean corpuscular hemoglobin (MCH) and shift work are consistently smaller in all the three linear methods, but larger in tmap. On the other hand, the effect size of time since previous relief is also larger in ADONIS, consistent with our previous comparison between envfit and tmap. Although smaller effect size of time since previous relief was observed with ANOSIM, it is statistically non-significant. We have already included these results in our revision (line 6-12, page 7, new supplementary Fig. S4).

Fig.2d is not referenced in the manuscript.

Response: We have revised the manuscript, and reference to Fig.2d has been indicated in the text (line 24, page 5).

**Reviewer #2**

However, it is not easy to understand each step of the algorithms and the underlying rational. The fig 1, overview of the workflow does not provide sufficient information about underlying steps. A detailed algorithm workflow would be definitely needed to aid understanding.

Response: Thank you very much for the comments. We have revised Fig. 1 and added a new figure (Fig. 7) to make both the overall workflow and algorithmic details of tmap clearer and more intuitive for better understanding of our method. First, we have revised Fig. 1 to focus on the overview of the pipeline of tmap, illustrating major steps of data processing and the results of microbiome data analysis. This will enable a streamlined and high-level understanding of using tmap to analyze high dimensional microbiome data and metadata into obtain biological insights, such as host covariate ranking and co-enrichment between covariates and taxa. Second, we have made a new figure (Fig. 7) for the key algorithms used within tmap. These two sub-figures now provide sufficient information about the algorithmic steps and their underlying rationale. In the schematic diagram of the Mapper algorithm (Fig. 7a), we have demonstrated carefully how the steps of filter, covering and clustering work together to transform a high dimensional dataset into an abstract network representation. And for the SAFE algorithm (Fig. 7b), we have illustrated the concept of subnetwork enrichment together with the calculation of their enrichment scores. This will allow the reader to understand how metadata or microbiome features can be mapped to TDA network for enrichment analysis to test for their associations with subnetwork structures, which can be descripted statistically by the proposed SAFE scores.

The same problem is observed for the method part. It lacks sufficient information.

Response: We have rewritten the method part with substantial improvement and sufficient information, as follows:

1.We have clearly explained each step of the Mapper algorithm with both descriptions of its algorithmic details and how microbiome data is used by the algorithm. Our revision now includes information on filter functions, covering and clustering (line 14- 27, page 14; line 1-15, page 15).

2.We have also elaborated on the SAFE algorithm, including the rationale underlying the algorithm (line 17-26, page 15), the steps of the calculation of SAFE scores (line 9- 16, page 16), and the use of SAFE scores for association analysis (line 22-29, page 4; line 1-4, page 5).

L28 on page 11: "filter/len function for mapping the original data points to their low dimentional coordinates, For instance, the first two principal coordinators of PCoA can be used as filters". What does "filters" mean? Are the first two sufficient? How to decide numbers of filters? What is the impact on the following steps? How robust it would be? Although authors said that detailed explanation and guideline are provided on line. It is still important to explain it clearly in the paper.

Response: The definition and use of filters in tmap have been thoroughly described in our revised manuscript (line 14-22, page 14), together with its illustration in the new Fig.7a. We have now proposed a strategy on the selection and evaluation of filters in our new Discussions (line 12-25, page 12) and Methods (line 6-17, page 21). This strategy can help us to decide how many and which filters (in the case of PCoA or PCA) to use in tmap, and to evaluate the impact on association analysis. We also have included other dimension reduction methods (t-SNE and UMAP) in our revised manuscript to assess the robustness of using different filters in the analysis of the FGFP dataset (line 28-29, page 12; line 1-5, page 13; line 18-26, page 21)

Page 12: Spatial analysis of functional enrichment (SAFE) algorithm for network enrichment analysis. The authors claimed that it can be applied to a target variable (or multiple target variables). It is unclear how it works for multiple target variables. The provided formula does not copy with multiple variables. L13 on page 13: "random shuffle is performed independently for each target variable when there are more than one". It is not a correct way, as target variables can be inter-correlated/confounding.

Response: Indeed, the SAFE algorithm works on single target variable (as illustrated in the new Fig. 7b). In the case of multiple target variables, the algorithm is applied to each of them independently. But the concern of inter-correlated or confounding variables can be addressed in tmap. As we have now discussed in our revised manuscript (line 22-29, page 13; line 1-2, page 14), tmap allows us to characterize interrelations between metadata or microbiome features, via co-enrichment network analysis, to help to clarify or determine whether these interrelations represent confounding effects or indicate biological interactions, given the context of studies (as demonstrated in our analysis of the FGFP, AGP and EMP microbiomes).

Page 8: enterotype-like stratification. The concept of enterotype has been abandoned in the field (see Knight et al. Cell Host Microbe. 2014 Oct 8;16(4):433-7). As shown in Fig 5, different groups can be formed based on the type of species under consideration. It is good to remove the concept of "enterotype".

Response: We agree that the concept of enterotype is arguable, and our results indeed extend beyond the traditional determination of enterotypes for microbiome stratification (line 17-24, page 9). To make our statement of the results more precise, we have removed “enterotype-like” in our revision. The subtitle and figure legend now read “Indepth stratification of …”. Also, we have now added a discussion on how our result revealed in-depth microbiome stratifications according to the change of a particular microbiome marker (line 16-20, page 9), and this reference was cited in our text.

L16: "a TDA network is constructed to represent the microbiome diversity". I don't understand it. How this network can represent the diversity?

Response: We apologize for the incorrect use of “microbiome diversity”. We have went through our manuscript to check for this incorrectness, and have corrected “microbiome diversity” to “microbiome variation” appropriately. Thus, the sentence reads as “a TDA network to represent the microbiome variation” (line 18, page 4), in which we mean that a TDA network captures the patterns of differences and similarities between samples in a high-dimensional microbiome space.

**2^nd^ round**

**Reviewer 1**

All my concerns have been addressed.

**Reviewer 2**

The authors have significantly improved the manuscript by providing more information on the algorithm and methods. However, there are still a few questions need further clarification.

Line7-18 on page 21: As TMAP deals with high-dimensional, complex microbiome data, filter function used only the top two PCs to lower dimension. If the first two PCs can sufficiently capture the variation, the data would not be considered as high dimensional.

“At last, all the PCs were ranked according to these weighted scores, to provide information

on how many and which PCs to select as filters in a final analysis”. But the authors still did not explain how to decide the number of PCs and which PCs to be selected.

Line 20: The robustness comparison was only done for the top 2 PCs. It is still a question whether the performance can be improved if more PCs can be used, particularly in the case of a highly complex microbiome data that the top 2 PCs only explained very small proportion of the variation.

Line 7 on page 6: “The poor performance of envfit and adonis”. But the performace of adonis was not bad. The Line 6 ”Average ROC AUC is 0.89 for adonis, compared to 0.95 for tmap, 0.55 for envift and 0.63 for ANOSIM. At line 12: “Average ROC AUC was 0.93 for adonis, compared to 0.98 for tmap, 0.82 for envfit and 0.73 for ANOSIM).

Based on above comparison, enfit had the worst performance. It is strange that authors evaluated the improvement of tmap by only comparing it to envfit. Line 13: “Similar improvement by tmap was observed when we varied the number of simulated metadata associated with the microbiome (tmap vs. envfit, Additional file 2: Fig. S2).

**Response to reviewers**

**Reviewer #2**

Line7-18 on page 21: As TMAP deals with high-dimensional, complex microbiome data, filter function used only the top two PCs to lower dimension. If the first two PCs can sufficiently capture the variation, the data would not be considered as high dimensional.

Response: Thank you very much for this comment about capturing microbiome variations in high dimensional space. We have substantially revised our manuscript to fully demonstrate and discuss the ability of tmap to capture and represent complex microbiome variations from high dimensional datasets, by the following revisions:

1.A large proportion of microbiome variance is uncaptured by the first two PCs of PCoA, e.g. 67.91%, 87.10% and 80.71% for the FGFP, AGP and EMP datasets, reflecting the high dimensional nature of their microbiome variations (line 7-11, page 12; Additional file 11: Fig. S11). Therefore, methods that are able to capture these

remained variances for microbiome analysis are necessary.

2 .We showed that tmap is able to recover microbiome variations from the original high dimensional space, even though they might be lost by the filters using dimension reduction methods (added Methods: line 19-28, page 22; line 1-23, page 23; added Discussion: line 12-21, page 12; Additional file 15: Fig. S15). Significant improvement of tmap in representing original microbiome variations than low dimensional projection space were shown for different methods and datasets (Additional file 16: Fig. S16, Additional file 17: Fig. S17) in our revised manuscript.

3.We further showed that tmap is robust to the number of PCs used as filters in capturing the original high dimensional distance information (tmap vs projection space, regression R2 of 0.640.10 vs 0.460.14, Additional file 17: Fig. S17, line 22-28, page 12; line 1-8, page 13). Therefore tmap is able to capture high dimensional microbiome variations even though only two PCs is used as filters, enabled by its clustering step and network representation.

With these added Methods, Discussion and new supplementary figures, we believe that the ability of tmap to analyze complex and high dimensional datasets is promising for large-scale microbiome studies, especially in comparison with currently available dimeson reduction methods.

“At last, all the PCs were ranked according to these weighted scores, to provide information on how many and which PCs to select as filters in a final analysis”. But the authors still did not explain how to decide the number of PCs and which PCs to be selected.

Response: We have added a revised workflow (Additional file 18: Fig. S18) for the evaluation and selection of the most suitable PCs as filters, in the case that a chosen set of target variables (metadata or microbiome features) are of interest. We believe that the revision (line 9-17, page 13, Additional file 18: Fig. S18) now clearly explain how to decide the number of PCs and which PCs to be selected as filters to optimize a TDA network according to the chosen target variables. As a result, given different target variables of interest, different PCs may be selected as the most informative filters (e.g. PC 8, 3 for Hemoglobin and PC 1, 2 for BSS in the FGFP dataset).

Line 20: The robustness comparison was only done for the top 2 PCs. It is still a question whether the performance can be improved if more PCs can be used, particularly in the case of a highly complex microbiome data that the top 2 PCs only explained very small proportion of the variation.

Response: We have included more PCs (up to 4 PCs) for tmap analysis when using different dimension reduction methods as filters. Although improved regression performance can be observed for the low dimensional projection space, tmap is robust to the number of PCs used (line 3-8, page 13, Additional file 17: Fig. S17, Additional file 14: Fig. S14). As we explained in the revised manuscript, tmap is able to capture and represent high dimensional distance information even though using filters of low dimensional projection that might not be able to capture those information (line 12-21, page 12; Additional file 15: Fig. S15). Therefore the robustness comparison done for the top 2 PCs should be able to reflect high dimensional variations not explained by the top 2 PCs.

Line 7 on page 6: “The poor performance of envfit and adonis”. But the performance of adonis was not bad. The Line 6 ”Average ROC AUC is 0.89 for adonis, compared to 0.95 for tmap, 0.55 for envift and 0.63 for ANOSIM. At line 12: “Average ROC AUC was 0.93 for adonis, compared to 0.98 for tmap, 0.82 for envfit and 0.73 for ANOSIM).

Response: We apologize for this imprecise description of the improvement by tmap over other methods, in detecting microbiome associated metadata. We have revised the manuscript to describe the significantly improved performance of tmap more precisely (line 23-29, page 5; line 1-3, page 6).

Based on above comparison, enfit had the worst performance. It is strange that authors evaluated the improvement of tmap by only comparing it to envfit. Line 13: “Similar improvement by tmap was observed when we varied the number of simulated metadata associated with the microbiome (tmap vs. envfit, Additional file 2: Fig. S2).

Response: In the revised manuscript, we have included simulations for all the methods in comparison, including envfit, adonis, ANOSIM and tmap. The result support our previous claims on the improvement by tmap over other methods under all the simulated scenarios when we varied the number of simulated metadata (line 10-11, page 6; Additional file 2: Fig. S2).

**3^rd^ round**

**Reviewer 2**

The authors have sufficiently addressed my comments.
